# Supplementary material for: Partial heart transplantation for pediatric heart valve dysfunction: A clinical trial protocol
Source: PLoS One. 2023 Feb 7;18(2):e0280163. doi: 10.1371/journal.pone.0280163 (PMC9904480; doi:10.1371/journal.pone.0280163)
Supplement: S1 Appendix — (DOCX) [file pone.0280163.s001.docx]

### **Appendix 1**

As required by OPTN policy, the MUSC Transplant Center follows a protocol for performing organ check-in as outlined below:

Procedure:

1. Organ check-in will be completed any time an organ is recovered outside the facility where the transplant will take place. The organ check-in must be completed upon arrival at the transplant hospital prior to opening the organ’s external transport container.
2. The OR charge nurse or designee is responsible for the organ check-in procedure:
   1. The OR charge nurse or designee will ensure that the donor UNOS ID, expected organ and laterality (if applicable) and ABO blood type are on the OR posting slip with the intended organ recipient name and medical record number.
   2. The OR charge nurse or designee will verify the received organ information with the OPO representative, Procuring Surgeon, or Courier designee.
   3. The OR charge nurse or designee will use the OPTN external label to confirm that the label contains the expected:
      1. Donor ID
      2. Organ type and laterality (if applicable)
3. If the donor ID, organ type or laterality information conflicts with the expected information, the OR charge nurse or designee will notify the OPO or the On-Call Transplant Coordinator who will then notify the OPO. This must occur as soon as possible, but within one hour of the determination.
4. The OR charge nurse or designee will ensure that all deceased donor organs are registered in the Organ Sign In/Sign Out Log located at the front desk of the OR, documenting:
   1. Date of arrival
   2. Time of arrival
   3. Organ type and laterality
   4. ABO
   5. UNOS ID
   6. Delivery personnel
   7. RN signature
   8. That the outer package and label are intact
5. The deceased donor organ is then dispensed to the intended transplant recipient operating suite for immediate transplantation. If the organ is not immediately transplanted, the organ will be stored in the temperature monitored OR refrigerator which is dedicated to transplant organ storage.
6. Organs that are not utilized for MUSC transplant and are picked up by an OPO representative or courier designee at a later time or date shall have the organ disposition documented within the OR Organ Sign In/Sign Out log.
7. The surgeon assumes the responsibility for the organ and its disposition after notification of arrival and will notify MUSC on-call transplant coordinator or OPO of disposition other than transplant within one hour of the determination.

As required by OPTN policy, the MUSC Transplant Center follows a protocol for performing pre-transplant verifications as outlined below.

1. Pre-Transplant Verification Prior to Organ Receipt

IF the recipient surgery will begin prior to organ receipt in the operating room, a pre-transplant verification must be conducted that meets all of the following requirements:

- 1. Two licensed health care professionals must participate in the verification
  2. The intended recipient must be present in the operating room
  3. The verification must occur either:
     1. Prior to the induction of general anesthesia (Bolus of midazolam in the PACU is not considered induction of general anesthesia).
     2. Prior to incision if the patient has been receiving continuous sedation prior to the arrival in the operating room (Bolus of midazolam in the PACU is not considered continuous sedation).
  4. At MUSC, the acceptable sources during the pre-transplant verification prior to organ receipt to verify all of the following information in the table are: expected donor IR, expected organ, expected donor blood type and subtype, recipient unique identifier, recipient blood type, expected donor and recipient are blood type compatible.
  5. This verification is documented in the recipient’s electronic medical record (EMR). In the EMR, the “performed” time is the actual time the pre-organ verification took place. The “verified” time is the time the licensed healthcare providers confirmed that the pre-organ verification took place at the documented performed time.

1. Pre-Transplant Verification Upon Organ Receipt

At the time of organ receipt in the operating room, a pre-transplant verification must be conducted with the following requirements:

- 1. The transplant surgeon and another licensed health care professional must participate in the verification.
  2. The intended recipient must be present in the operating room.
  3. The verification must occur after the organ arrives in the operating room but prior to anastomosis of the first organ.
  4. At MUSC, the acceptable sources during the pre-transplant verification upon organ receipt to verify all of the following information in the table are: expected donor IR, expected organ, expected donor blood type and subtype, recipient unique identifier, recipient blood type, expected donor and recipient are blood type compatible.
  5. This verification is documented in the recipient’s electronic medical record (EMR). In the EMR, the “performed” time is the actual time the organ verification took place. The “verified” time is the time the licensed healthcare provider and the transplanting surgeon confirmed that the organ verification took place at the documented performed time.
