# Supplementary material for: Partial heart transplantation for pediatric heart valve dysfunction: A clinical trial protocol
Source: PLoS One. 2023 Feb 7;18(2):e0280163. doi: 10.1371/journal.pone.0280163 (PMC9904480; doi:10.1371/journal.pone.0280163)
Supplement: S2 Appendix — (DOCX) [file pone.0280163.s002.docx]

**Appendix 2**

This study involves greater than minimal risk with the potential for direct benefit. In conventional heart transplantation, the most serious risks are graft failure, cardiac allograft vasculopathy, infection and acute rejection. Together these account for the majority of deaths after conventional heart transplantation. The relative importance of these causes of death vary over time post-transplant.

The risks for a partial heart transplantation are lower than orthotopic heart transplantation. There is no risk for transplant coronary artery disease or myocardial dysfunction. We expect that graft failure and cardiac allograft vasculopathy will not be important risks after partial heart transplantation. The reason for this is that conventional heart transplants perform active work to sustain hemodynamics, whereas the partial heart transplant merely serves a structural function. There is no need for repeat cardiac catheterizations with myocardial biopsy for invasive assessment of hemodynamics and surveillance of rejection and transplant coronary artery disease. There is no risk of tricuspid regurgitation due to tricuspid valve apparatus damage from recurrent myocardial biopsies.

There is a risk that subjects could deteriorate while on the waiting list for partial heart transplant. Given that patients listed for partial heart transplantation will generally be expected to have preserved ventricular function, we would anticipate this risk will be less than or similar to that incurred by similar-aged children on the waiting list for orthotopic heart transplantation.

Risk of death: we expect the operative risk of partial heart transplantation to be similar to the risks from homograft valve replacement. We expect the long-term risks to be lower than the long-term risks from conventional heart transplantation and far lower than the long-term risks from re-operations for homograft implant exchanges.

Risks of **general anesthesia** include nausea, vomiting, blood vessel injury, nerve injury, lung injury, heart attack, allergy to drugs, brain damage, and death.

Risks of **partial heart transplant** are expected to be similar to the risks of standard valve replacement and less than the risks of full heart transplant.

1. The most serious risks are scheduling issues related to donor heart availability and rejection of the transplanted valve. The risk of rejection is theoretically lower because less tissue is being transplanted compared to a full heart transplant. The risk of death during and after the operation is similar to the risks for standard valve replacement. There is no risk of disease developing in the blood vessels that supply the heart and no risk of heart muscle dysfunction directly resulting from valve replacement.
2. The surgery will result in a permanent scar in the middle of the chest. This is the same scar that would result from a standard valve replacement or full heart transplant. This scar may require medical follow-up after surgery and may restrict you from exercise and/or sports participation in the future.
3. Open heart surgery involves the risk of death, brain damage, or other organ damage such as kidney injury.
4. The need for blood transfusion during surgery with the risk of infection from the blood or a reaction to the blood.
5. Patients receiving a partial heart transplant may also become sensitized. This will decrease the donor pool if the patient ever requires a heart transplant.
6. There is a risk of loss of confidentiality.
7. There may also be additional risks that are as yet unknown to us.

Risks of **immunosuppressive medications** are the same as the risk of using these medications after full heart transplant because the same medications are used. These risks include:

1. An increased risk of infections.
2. An increased risk of blockage of the coronary arteries.
3. Perhaps an increased risk of cancer or tumors after several years of these medicines.
4. Potential kidney toxicity such as high blood pressure or loss of salts in the urine.

The medication risks primarily include daily burden of taking the medications and infection. Because these medications prevent the immune system from attacking the transplanted valve, they will also prevent the immune system from fighting infection. Serious long-term effects of these medications individually and in combination with each other include permanent kidney injury, development of cancer, and early development of heart and blood vessel disease. The following includes the risks of each medication.

1. Tacrolimus
   1. Very common effects occurring in more than 30% of patients: tremor (uncontrollable shaking), high blood pressure, low phosphate levels, increased creatinine (a marker of kidney injury), infection, headache, diarrhea, nausea, swelling of the arms and legs, constipation, urinary tract infection, low magnesium levels, lack of energy, abdominal pain, other bodily pain, difficulty sleeping, high cholesterol levels, high potassium levels, and low red blood cell levels
   2. Very common adverse effects occurring in more than 10% but less than 30% of patients: vomiting, indigestion, fever, joint pain, back pain, diabetes that may require insulin to control high blood sugar, burning or prickling sensations, low potassium levels, low blood sugar levels, shortness of breath, dizziness, chest pain, increased cough, swelling, skin rash, itching, low white blood cell levels
2. Mycophenolate
   1. Very common effects occurring in more than 10% of patients: high blood sugar levels, high cholesterol levels, low magnesium levels, shortness of breath, back pain, increased blood nitrogen levels, low white blood cell levels, fluid around the lung, urinary tract infection, increasing frequency of cough, low calcium levels, high blood pressure, abdominal pain, swelling of the arms and legs, low red blood cell levels, fever, nausea, high potassium levels, diarrhea, infection from bacteria, fungus, or virus, and headache
   2. Common effects occurring in more than 1% but less than 10% of patients: melanoma, a type of skin cancer, other cancers, lymphoma (cancer of the immune system), gastrointestinal bleeding
   3. Rare effects occurring in less than 0.1% of patients:
      1. Progressive multifocal leukoencephalopathy, a serious neurological disorder
3. Prednisone, or methylprednisolone, both of which are steroids
   1. Very common effects occurring in more than 10% of patients: indigestion, nausea, high blood pressure, mood swings, difficulty sleeping, cataracts (clouding of the lens of the eye), slow wound healing, bruising, skin rash, growth suppression in children, swelling of the face, increased risk of bone fracture, high blood sugar
   2. Rare side effects occurring in less than 1% of patients: muscle weakness, severe nausea, fever, difficulty breathing, pancreatitis (inflammation of the pancreas)

Risks of **blood collection** associated with drawing blood include momentary discomfort and/or bruising. Infection, excess bleeding, clotting, or fainting is possible, although unlikely.

Risks of **data collection** include loss of confidentiality. Data collected as part of the study will be available only to study investigators and the research team.

Other potential risks:

Data collected as part of the study protocol will be available only to study investigators. Confidentiality will be respected and maintained at all times. The investigators will obtain permission from the primary cardiologist or cardiac surgeon to speak with parents/guardians about the study to protect their privacy.

Pediatric cardiac surgical procedures result in significant parental anxiety and distress [22]. The study investigators are familiar with interacting with parents in this situation and will be cognizant of parental needs. No aspects of this study are expected to have a deleterious effect on parents’ psychological well-being.
