# Supplementary material for: Partial heart transplantation for pediatric heart valve dysfunction: A clinical trial protocol
Source: PLoS One. 2023 Feb 7;18(2):e0280163. doi: 10.1371/journal.pone.0280163 (PMC9904480; doi:10.1371/journal.pone.0280163)

**Medical University of South Carolina
CONSENT TO BE A RESEARCH SUBJECT**

**TITLE OF RESEARCH:** Partial Heart Transplantation for Severe Pediatric Semilunar Heart Valve Dysfunction

# SUMMARY

Your child is being offered participation in a research study. Research studies are voluntary and include only people who choose to take part and meet certain criteria.

- The research study is not sponsored by any corporation or company.
- This is a research study to evaluate a new surgery designed to deliver growing heart valve replacements. If you agree to participate, your child will have surgery on a heart valve that does not work correctly. This heart valve will be removed and replaced with a heart valve implant. The current state of the art are heart valve implants that are made from human valves from deceased donors (“**homografts**”), animal tissues (“**biological valves**”) or metal (“**mechanical valves**”), none of which will grow with your child over time. A **conventional heart transplant** would grow with your child but this would involve replacing the entire heart with donor tissue. In contrast, the new heart valve used in this research study will come from a freshly donated human heart that could otherwise be used for a conventional heart transplant. Unlike a conventional heart transplant, only the valve will be transplanted from the donor heart. Therefore, we have called the new surgery a “**partial heart transplant.**” The objective of this operation is to combine the benefits of heart valve replacement and heart transplantation in order to deliver heart valve implants that grow with your child in order to minimize the need for re-operations as your child grows.
- The reasonably foreseeable risks and discomforts are the same as for heart valve replacement and heart transplantation. These include stroke, nerve damage (e.g. recurrent laryngeal nerve or phrenic nerve), heart attack, heart valve dysfunction, heart rhythm dysfunction, organ damage (e.g. kidneys or lungs), bleeding, infection, and death.
- After the operation, your child will need to take medications to prevent that his/her immune system from attacking the donor cells in the new valve. If these medications are stopped, then the valve would stop growing and turn from a partial heart transplant into a homograft, which is the current standard of care. The size and function of the new valve will be evaluated with ultrasound, an examination with sound waves.
- There are risks to the surgery that are described in this document. Some of the risks include specific risks of general anesthesia, need for additional surgery if the new heart valve fails, and side effects of medications needed after surgery.
- The total length of the study is 1 year. Following surgery, your child’s care will be determined by your child’s clinical team. During the study, the research team will collect data from the medical records that are kept for your clinical care. Your child can stop participating in the research study at any time.
- If the surgery is superior to the current standard of care, your child may benefit from participating in the study, but this is not guaranteed. Possible benefits include minimizing the need for future interventions on the replaced heart valve, better function of the replaced heart valve over time, and better survival. Alternatives to participation in this research study may include conventional heart valve surgery, medical treatments, heart transplantation, or palliative care.
- Alternatives to participation in this research study may include conventional heart transplantation, conventional heart valve surgery, medical treatments, or palliative care.

If you are interested in learning more about this study, please continue to read below.

# PURPOSE OF THE RESEARCH

It is unknown if a new heart surgery, called “partial heart transplantation”, would benefit children with unrepairable semilunar heart valve dysfunction. The purpose of this study is to evaluate the safety and effectiveness of the new heart surgery. Your child is being offered participation in this study because your child has unrepairable semilunar heart valve dysfunction (e.g. aortic valve dysfunction, or truncus arteriosus valve dysfunction).

This research study is a pilot study. A pilot study is a small-scale test of a new procedure.

There will be no new drugs or new devices used in this research. The new part of this surgery is that the valve will come from a fresh donor heart and that immune suppression will be used to allow the valve to grow with your child over time. A partial heart transplantation will require medical care after surgery that is more similar to the care after a full heart transplant than the care after a heart valve replacement with a conventional implant. The anticipated benefit is that the growing valve will minimize the need for re-operations on the valve and avoid the inevitable myocardial dysfunction that occurs following full heart transplant.

Please read this consent form carefully and take your time making your decision. As your study doctor or study staff discusses this consent form with you, please ask him/her to explain any words or information that you do not clearly understand. You are being asked to allow your child to participate in this study because your child has unrepairable semilunar heart valve dysfunction. The investigators in charge of this study at MUSC are Dr. Rajab and Dr. Costello. The study is being done at 1 site. Approximately 5 people will take in this study.

# PROCEDURES

If you agree to allow your child to participate in this study, the following will happen:

To see if your child is eligible for the study:

The research team will check your child’s medical records to gather information about your child’s heart function and anatomy. Any pre-operative tests and procedures will be determined by your clinical team.

If your child is eligible for the study:

- Your child’s clinical team will make the decision whether your child will be treated with a partial heart transplant according to the study protocol, a heart transplant, heart valve replacement or another treatment.
- If your child is eligible for the study, your child will be listed for partial heart transplant. Your child would then need to wait for a donor heart to become available. This wait may be weeks or months.
- When a donor heart becomes available, you will be notified by your child’s clinical team. Therefore, you will need to carry a transplant pager and be able to arrive expediently at the hospital.
- There are some circumstances where your child may be removed from the research study before or after surgery. If your child’s heart or heart valve is not functioning well, a decision may be made to offer another treatment, such as a standard valve replacement or a conventional full heart transplant.

Surgical procedures:

- The donor heart will be recovered and the new heart valve will be removed from this heart by the clinical team.
- Your child will be scheduled for an emergent operation once the donor heart valve is deemed acceptable.
- Your child will have **general anesthesia** during surgery. This is a state of unconsciousness, which is carefully controlled by the anesthesiologist with a mixture of very potent drugs, to prevent or lessen pain. This is the same type of anesthesia that is used for other types of heart surgery.
- Your child will have a **partial heart transplant** using the donated heart valve to replace the dysfunctional heart valve. This is an experimental procedure.
- Expected hospital stay will range from weeks to months.
- The medical care after surgery is performed by your child’s clinical team.
- In the case that there is a problem with the valve during or immediately after surgery, a standard valve replacement will be performed. This type of re-operation could similarly be required after a standard heart valve replacement.

After surgery

- Your child will be treated by the clinical team as any other child with an heart transplant or heart valve replacement.
- The clinical team will admit your child to the pediatric cardiac intensive care unit (ICU) in the hospital. This unit has specialized heart doctors and nurses who take care of children after heart surgery. They will closely monitor your child. This will be the same as what is currently done after a heart transplant.
- The clinical team will perform tests and procedures as they see fit. This typically includes echocardiograms and blood draws. The results will be recorded as part of the study.
- The clinical team will treat your child with immunosuppressive medication. These are drugs that suppress the body's immune response and prevent the immune system from injuring the transplanted valve. The type of medication will be the same as for a full heart transplant and these medications are known to be generally safe. The type and amount of drug given are determined by the clinical team and may change if side effects develop. Some form of immunosuppression will likely be continued until the transplanted valve is replaced later on in life.
- The clinical team will decide when your child will be able to leave the hospital. These are the same standard criteria as for a full heart transplant or heart valve replacement.
- The clinical team will decide how long your child will need to stay near the hospital. Typically this is up to 4 weeks after discharge in case of emergencies. This is the same as for full heart transplant and is not for a research-related purpose. If your child lives outside the Charleston area, your child will be able to leave the Charleston area when the pediatric heart doctor decides it is safe for your child to go home.
- The clinical team will schedule clinic appointments as needed after your child leaves the hospital. This is expected to follow same schedule as for heart transplant. These clinical follow-up visits typically occur every 4 weeks during the first 6 months and then every 6 months after that. Your child will be taken care of by a pediatric cardiologist. There will no additional visits or tests for research purposes. If your child does not follow-up at MUSC, we will obtain relevant information from your child’s pediatric cardiologist.
- The research team will review the medical records of your child for one year after the operation. If your child’s care is with providers who are not part of the MUSC network, the research team will obtain the medical records of your child from those providers.

# DURATION

Participation in the study will continue for one year. During this time the research team will review the medical records for your child.

# RISKS AND DISCOMFORTS

Risks of **general anesthesia** include nausea, vomiting, blood vessel injury, nerve injury, lung injury, heart attack, allergy to drugs, brain damage, and death.

Risks of **partial heart transplant** are expected to be similar to the risks of standard valve replacement and less than the risks of full heart transplant.

- The most serious risks are scheduling issues related to donor heart availability and rejection of the transplanted valve. The risk of rejection of the partial heart transplant is theoretically lower than a conventional heart transplant because less tissue is being transplanted. Furthermore, in patients who have received a total heart transplant, the aortic and pulmonary valves continue to function well during episodes of rejection. The risk of death during and after the operation is similar to the risks for standard valve replacement.
- The surgery will result in a permanent scar in the middle of the chest. This is the same scar that would result from a standard valve replacement or full heart transplant. This scar may require medical follow-up after surgery.
- Open heart surgery involves the risk of death, brain damage, or other organ damage such as kidney injury.
- The surgery will involve blood transfusion, with the risk of infection from the blood or a reaction to the blood.
- There may also be additional risks that are as yet unknown to us.

Risks of **delayed treatment** include clinical deterioration while on the waiting list that are less than or similar to that incurred by patients waiting for heart transplants.

Risks of **immunosuppressive medications** are the same as the risk of using these medications after full heart transplant. These risks include:

- An increased risk of infections.
- Perhaps a slightly increased risk of cancer or tumors after receiving several years of these medicines.
- Risk of kidney insufficiency such as high blood pressure or loss of salts in the urine.
- Risk of developing diabetes
- These medications are called tacrolimus, mycophenolate, and prednisone. The medications are routinely used by pediatricians for a variety of conditions, and the risks of these medications are well-known. The medication risks and discomforts include the daily burden of taking the medications. Because these medications prevent the immune system from attacking the transplanted valve, they will also decrease the immune system’ ability to fight infection. Serious long-term effects of these medications individually and in combination with each other include permanent kidney injury, development of cancer, and early development of heart and blood vessel disease. The following includes the risks of each medication.

Tacrolimus

- Very common effects occurring in more than 30% of patients: tremor (uncontrollable shaking), high blood pressure, low phosphate levels, increased creatinine (a marker of kidney injury), infection, headache, diarrhea, nausea, swelling of the arms and legs, constipation, urinary tract infection, low magnesium levels, lack of energy, abdominal pain, other bodily pain, difficulty sleeping, high cholesterol levels, high potassium levels, and low red blood cell levels.
- Common adverse effects occurring in more than 10% but less than 30% of patients: vomiting, indigestion, fever, joint pain, back pain, diabetes that may require insulin to control high blood sugar, burning or prickling sensations, low potassium levels, low blood sugar levels, shortness of breath, dizziness, chest pain, increased cough, swelling, skin rash, itching, low white blood cell levels.

Mycophenolate

- Common effects occurring in more than 10% of patients: high blood sugar levels, high cholesterol levels, low magnesium levels, shortness of breath, back pain, increased blood nitrogen levels, low white blood cell levels, fluid around the lung, urinary tract infection, increasing frequency of cough, low calcium levels, high blood pressure, abdominal pain, swelling of the arms and legs, low red blood cell levels, fever, nausea, high potassium levels, diarrhea, infection from bacteria, fungus, or virus, and headache
- Common effects occurring in more than 1% but less than 10% of patients: melanoma, a type of skin cancer, other cancers, lymphoma (cancer of the immune system), gastrointestinal bleeding
- Rare effects occurring in less than 0.1% of patients: Progressive multifocal leukoencephalopathy, a serious neurological disorder

Steroids, typically prednisone, or methylprednisolone

- Common effects occurring in more than 10% of patients: indigestion, nausea, high blood pressure, mood swings, difficulty sleeping, cataracts (clouding of the lens of the eye), slow wound healing, bruising, skin rash, growth suppression in children, swelling of the face, increased risk of bone fracture, high blood sugar
- Rare side effects occurring in less than 1% of patients: muscle weakness, severe nausea, fever, difficulty breathing, pancreatitis (inflammation of the pancreas)

Side effects of medications may be mild, moderate, or severe. Some of these side effects can be serious, long lasting, or permanent. It is not possible to tell which side effect will affect your child or how mild or severe the side effect might be. You should notify your child’s study doctor and clinical doctor right away about any side effects, problems, or unusual experiences your child may have while taking the medication involved with this study. This will decrease the chance that the side effects continue or become worse. Sometimes there are other medications that can be used to lessen the side effects or make your child more comfortable.

Risks of **blood collection** associated with drawing blood include momentary discomfort and/or bruising. Infection, excess bleeding, clotting, or fainting is possible, although unlikely.

Risks of **data collection** include loss of confidentiality. Data collected as part of the study will be available only to study investigators and the research team.

# MEDICAL RECORDS AND/OR CERTIFICATE OF CONFIDENTIALITY

If your child is an MUSC patient your child has an MUSC medical record. If your child has never been an MUSC patient, a MUSC medical record will be created for the purposes of this study. Results of research tests or procedures will be included in your child’s MUSC medical record. All information within your child’s medical record can be viewed by individuals authorized to access the record. We will make every effort to keep confidential all research information in the medical record that identify you to the extent allowed by law.

# BENEFITS

If the surgery is successful in treating your child’s heart valve problem with fewer side effects than the current standard therapy, your child may benefit from participating in the study; however, this cannot be guaranteed. Potential benefits include fewer reoperations for heart valve replacement and improved survival. It is hoped that the information gained from the study will help in the treatment of future patients with conditions like your child’s and will help the researcher learn more about how to replace heart valves.

# COSTS

Routine medical care for your child’s condition will be charged to you or your child’s insurance company. The costs for the operation and medications will be charged to your insurance company. You may wish to contact your child’s insurance company to discuss this further. It is possible that your child’s insurance company will refuse to pay for the costs associated with research study participation. Please ask Dr. Taufiek Konrad Rajab if you would like to know more about which tests and studies are being done solely for research purposes.

# PAYMENT TO PARTICIPANTS

Your child will not be paid for participating in this study.

# ALTERNATIVES

Your child does not have to participate in this study to have his/her condition treated. If you choose not to participate in this study, your child could receive other standard treatments for your condition. The standard therapies for your condition are:

1. Ross operation: this means cutting out a healthy valve from your child’s heart, replacing the diseased valve with this healthy valve and using an artificial valve to replace the valve that was cut out. Risks of the Ross operation include stroke, nerve damage, heart attack, heart valve dysfunction, heart rhythm dysfunction, requirement for further operations to the valves, organ damage, bleeding, infection, and death.
2. A valve replacement: this means using a valve from dead human tissue, dead animal tissue, or made from metal. This is called heart valve replacement with a conventional implant. Risks of this operation include stroke, nerve damage, heart attack, heart valve dysfunction, heart rhythm dysfunction, organ damage, bleeding, infection, and death.
3. A heart transplant: this means removing your child’s entire heart and replacing it with a live heart from a human. Risks of this operation include stroke, nerve damage, heart attack, heart valve dysfunction, heart rhythm dysfunction, failure of the heart transplant, immune rejection, organ damage, bleeding, infection, malignancy and death.

# DATA SHARING

Information about your child (including identifiable private information and/or any identifiable biospecimens) may have all of you child’s identifiers removed and used for future research studies or distributed to other researchers for future research without additional informed consent from you, your child or your legally authorized representative.

# DISCLOSURE OF RESULTS

You will be told about any relevant clinical information from the clinical team.  The researchers will not be obtaining any information that would not already be in your child's medical record.

# Significant New Findings

If there are significant new findings during the course of the study, you will be notified*.*

# ClinicalTrials.Gov

A description of this clinical trial will be available on <http://www.ClinicalTrials.gov>, as required by U.S. Law. This Web site will not include information that can identify you. At most, the Web site will include a summary of the results. You can search this Web site at any time.

# Authorization to Use and Disclose (Release) Medical Information

As part of this research study, your child’s study doctor and his/her research team will keep records of your child’s participation in this study.

The health information MUSC may use or disclose (release) for this research study includes information in your child’s medical record, results of physical exams, medical history, lab tests or certain health information indicating or relating to your condition.

Your child’s study doctor and his/her research team will use and disclose (release) your child’s health information to conduct this study. The health information listed above may be used by and/or disclosed (released) to the following, as applicable:

- The sponsor of the study including its agents such as data repositories or contract research organizations monitoring the study;
- Other institutions and investigators participating in the study;
- Data Safety Monitoring Boards;
- Accrediting agencies;
- Clinical staff not involved in the study whom may become involved if it is relevant;
- Parents of minor children if less than 16 years old. Parents of children 16 years old or older require authorization from the child; or
- Health insurer or payer in order to secure payment for covered treatment;
- Federal and state agencies and MUSC committees having authority over the study such as:
- The Institutional Review Board (IRB) overseeing this study; Committees with quality improvement responsibilities; Office of Human Research Protections; Food and Drug Administration; National Institutes of Health or Other governmental offices, such as a public health agency or as required by law.

Those persons who receive your child’s health information may not be required by Federal privacy laws (such as the Privacy Rule) to protect it and may share your child’s information with others without your permission, if permitted by laws governing them. You do not have to sign this consent form. If you choose not to sign, it will not affect your child’s treatment, payment or enrollment in any health plan or affect your child’s eligibility for benefits. However, you will not be allowed to be a participant in this research study.

You will be given a copy of this consent form. Your authorization will expire at the conclusion of this study or, if you are participating in a study designed for the development of a drug or device, your authorization will remain in effect until the drug or device is approved by the FDA or until the company’s application to study the drug/device is withdrawn. You have the right to withdraw your agreement at any time. You can do this by giving written notice to your child’s study doctor. If you withdraw your agreement, you will not be allowed to continue participation in this research study. However, the information that has already been collected will still be used and released as described above. You have the right to review your child’s health information that is created during your child’s participation in this study. After the study is completed, you may request this information.

Your child’s health information will be used or disclosed when required by law. Your child’s health information may be shared with a public health authority that is authorized by law to collect or receive such information for the purpose of preventing or controlling disease, injury or disability and for conducting public health surveillance, investigations or interventions. No publication or public presentation about the research study will reveal your child’s identity without another signed authorization from you.

If you have questions or concerns about this Authorization or your child’s privacy rights, please contact MUSC’s Privacy Officer at (843) 792-8740.

Regulations require that you be given a copy of the MUSC Notice of Privacy Practices (NPP) describing the practices of MUSC regarding your child’s health information. One can be found at the end of this form.

Results of this research will be used for the purposes described in this study. This information may be published, but your child will not be identified. Information that is obtained concerning this research that can be identified with your child will remain confidential to the extent possible within State and Federal law. The investigators associated with this study, employees of the sponsor, and the MUSC Institutional Review Board for Human Research will have access to identifying information. All records in South Carolina are subject to subpoena by a court of law.

In the event that your child is injured as a result of participation in this study, you should immediately take your child to the emergency room of the Shawn Jenkin’s Children’s Hospital, or in case of an emergency go to the nearest hospital, and tell the physician on call that your child is in a research study. They will call your child’s study doctor who will make arrangements for your child’s treatment. If the study sponsor does not pay for your child’s treatment, the Shawn Jenkin’s Children’s Hospital and the physicians who render treatment to your child will bill your child’s insurance company. If your child’s insurance company denies coverage or insurance is not available, you will be responsible for payment for all services rendered to your child.

Your child’s participation in this study is voluntary. You may refuse to allow your child to take part in or stop taking part in this study at any time. You should call the investigator in charge of this study if you decide to do this. Your decision not to allow your child to take part in the study will not affect your or your child’s current or future medical care or any benefits to which you are entitled.

The investigators and/or the sponsor may stop your child’s participation in this study at any time if they decide it is in your child’s best interest. They may also do this if your child does not follow the investigator’s instructions.

# Volunteers Statement

I have been given a chance to ask questions about this research study. These questions have been answered to my satisfaction. If I have any more questions about my child’s participation in this study or study related injury, I may contact Dr. Taufiek Rajab at 843-792-3361 or Dr. John Costello at 843-792-3361. I may contact the Medical University of SC Patient and Family Care Liaison (843) 792-5555 concerning medical treatment.

If I have any questions, problems, or concerns, desire further information or wish to offer input about my child’s rights as a research subject in this study, I may contact the Medical University of SC Institutional Review Board for Human Research IRB Manager or the Office of Research Integrity Director at (843) 792- 4148. This includes any questions about my rights as a research subject in this study.

I agree for my child to participate in this study. I have been given a copy of this form for my own records.

If you wish to participate, you should sign below.

________________________________

Signature of Person Obtaining Consent Date *Name of Participant

___________________________________________________

Name of Mother / Guardian (Please print)

___________________________________________________

Signature of Mother Date

___________________________________________________

Name of Father / Guardian *(Please print)*

___________________________________________________

Signature of Father Date

Participant’s Personal Representative (if applicable):

___________________________________________________

Name of Personal Representative (*Please print)*

___________________________________________________

Signature of Personal Representative Date

Relationship:  ___ Spouse       ___ Parent               ___Next of Kin

  ___Legal Guardian* ____ DPOA for Healthcare*

___________________________________________________

Name of investigator obtaining consent *(Please print)*

___________________________________________________

Signature of Investigator Date


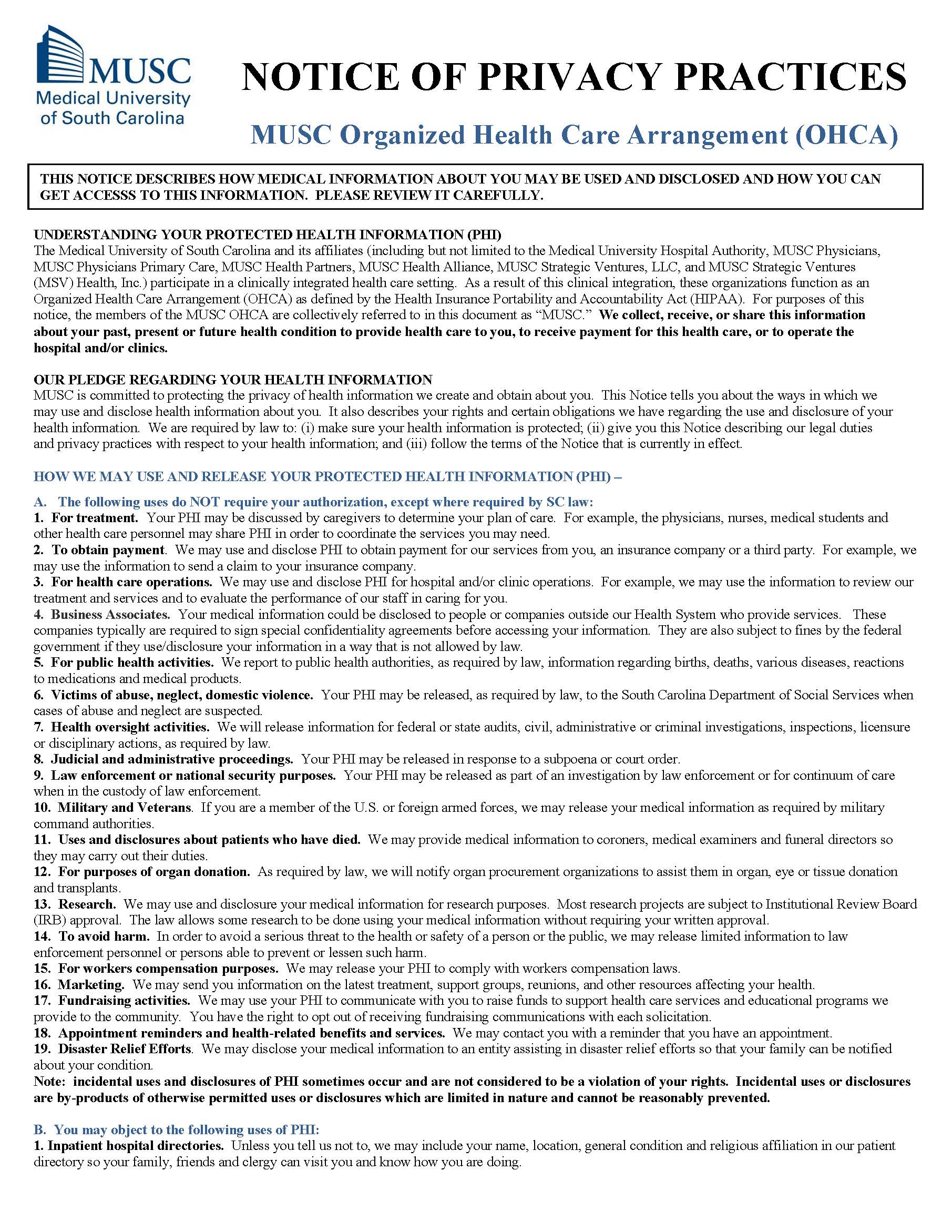


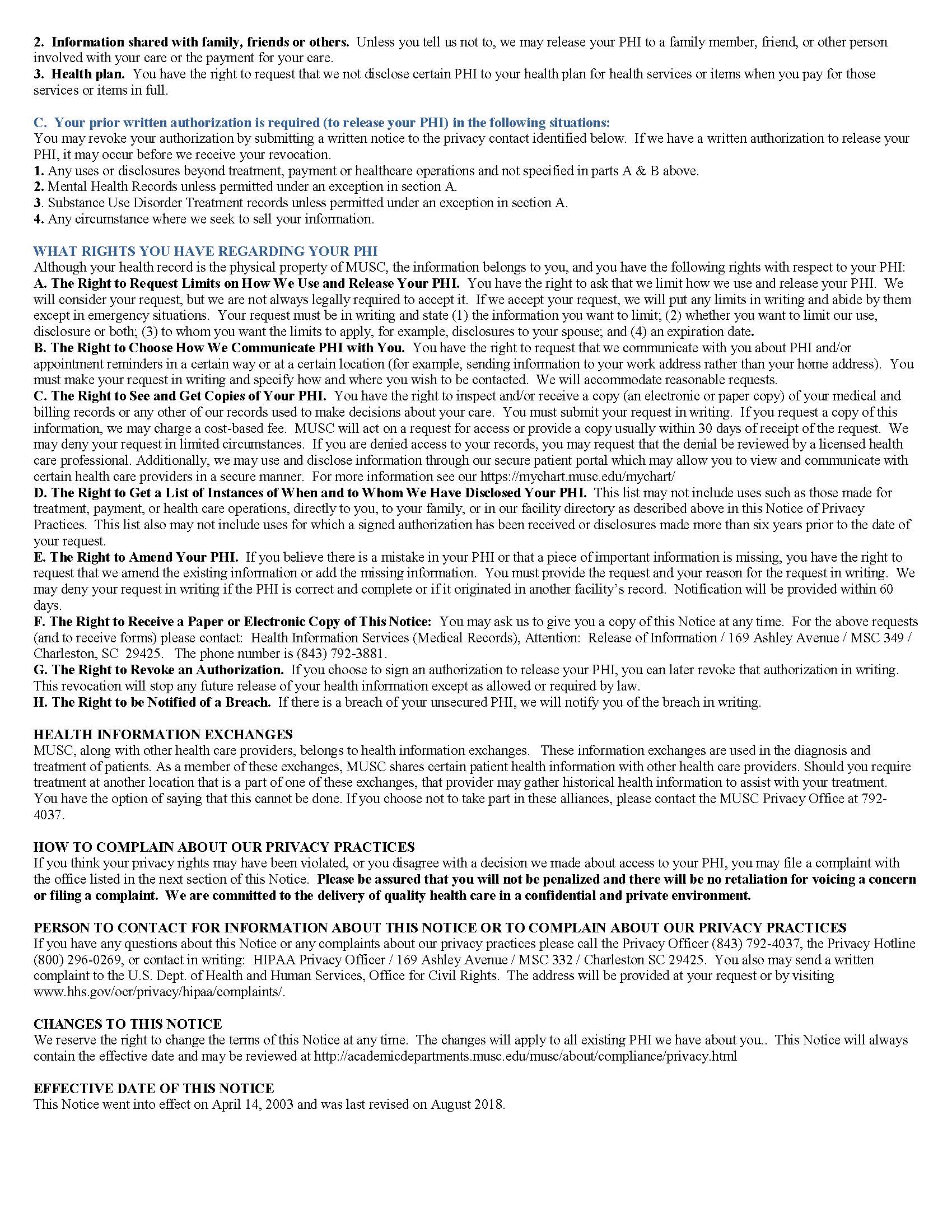

Supplement: S3 Appendix — (DOCX) [file pone.0280163.s003.docx]
