# Supplementary material for: Partial heart transplantation for pediatric heart valve dysfunction: A clinical trial protocol
Source: PLoS One. 2023 Feb 7;18(2):e0280163. doi: 10.1371/journal.pone.0280163 (PMC9904480; doi:10.1371/journal.pone.0280163)
Supplement: S1 File — (PDF) [file pone.0280163.s005.pdf]

Date: Monday, January 2, 2023 2:24:56 PM

Print Close

Pro00114653: Partial Heart Transplantation for Semilunar Heart Valve Dysfunction - Taufiek Rajab

View: Study Identification - Identification

Study Identification Information

This is the first step in your Human Research Application. You will automatically be guided to the appropriate forms needed to complete your submissions.

1.0 * **Full Title:**
Enter the full study title
Partial Heart Transplantation for Severe Pediatric Semilunar Heart Valve Dysfunction

2.0 * **Short Title:**
Enter a short descriptive title for this study (65 characters maximum):
Partial Heart Transplantation for Semilunar Heart Valve Dysfunction

3.0 * **Briefly describe the scientific or scholarly rationale:**
(i.e. purpose of research)
Congenital heart defects are the most common type of birth defects in humans. Treatment of congenital heart defects frequently involves heart valve replacement. However, heart valve replacement in neonates and infants is associated with substantial long-term morbidity and mortality. A major cause for this is that they always outgrow their heart valve implants with somatic growth. Additionally, over time, heart valve implants uniformly experience deterioration in function, due to the development of stenosis, regurgitation, or both. Therefore, such patients undergo recurrent reoperations for heart valve implant exchanges with successively larger implants. Each reoperation is associated with substantial risk of complications including death. As a result, there is an urgent clinical need for growing heart valve replacements.

The status quo is that no growing heart valve replacements are clinically available. We propose to deliver heart valve implants that will grow with neonates, infants and young children and maintain function over time through a novel operation called “partial heart transplantation”. This procedure involves surgical replacement of the unrepairable heart valve with a fresh heart valve allograft. Immune suppression will be required, similar to that used for a conventional heart transplant. The risks of immune suppression can be controlled, as shown by children with conventional heart transplants who live healthy and happy lives. Once the recipient child has grown, the transplanted heart valve can be replaced with an adult size mechanical prosthesis at which time the immunosuppression may be discontinued. Partial heart transplantation should be far superior to homograft valve replacement from a surgical perspective given the potential for valve growth and superior function. This is significant because it will spare young children with unrepairable heart valves from morbid reoperations for successive implant exchanges.

We propose a single-center, nonrandomized single arm pilot trial in neonates, infants and young children who require semilunar valve replacement. In this “first in man” trial, we seek to determine whether valve replacement using partial heart transplant is feasible and safe. Primary aims are survival at one year and five years following the procedure. We hypothesize that, when compared to historical controls

who have undergone homograft valve replacement, those undergoing partial heart transplantation will have equal or superior survival. Secondary aims are to assess growth and function of the transplanted valve. We hypothesize that when compared to historical controls who have undergone conventional valve replacement, those undergoing partial heart transplantation will have valve growth that corresponds with somatic growth and superior valve function 1 year following the procedure.
Up to five patients will be enrolled in this trial over three years.

4.0 * **Brief Study Summary**

Non-scientific description of the research study, using 3 to 10 sentences:

Statements such as "see protocol" are not acceptable.

Note: Text entered in the Brief Study Summary field will be used to describe your study at www.SCresearch.org, an online directory designed to facilitate recruitment, if inclusion on this site is indicated later in the application.

The long-term goal of our research initiative is to develop a new valve replacement option for neonates, infants and young children. Our central hypothesis is that transplantation of a freshly isolated heart valve will be associated with superior outcomes compared to currently available options, including preserved cadaver valves, bioprosthetic tissue valves, or mechanical valves. We have named this new operation "partial heart transplantation".

We propose a single-center, nonrandomized single arm pilot trial of "partial heart transplantation" in neonates, infants and young children who require semilunar heart valve replacement. In this “first in man” trial, we seek to determine whether valve replacement using partial heart transplant is feasible and safe. Primary aims are survival one year and five years following the procedure. We hypothesize that, when compared to historical controls who have undergone homograft valve replacement, those undergoing partial heart transplantation will have equal or superior survival one year and five years following the procedure. Secondary aims are to assess growth and function of the transplanted valve. We hypothesize that when compared to historical controls who have undergone conventional valve replacement, those undergoing partial heart transplantation will have valve growth that corresponds with somatic growth and superior valve function 1 year following the procedure. Up to five patients will be enrolled in this trial over three years.

5.0 * **Is this a pilot study?**

● **Yes** ○ No

Pro00114653: Partial Heart Transplantation for Semilunar Heart Valve Dysfunction - Taufiek Rajab

View: Study Identification - Institutional Review Board

Institution

1.0 * **Select the appropriate Institutional Review Board (IRB) for review**:

- ○ AnMed Health Medical Center
- ● **Medical University of South Carolina**
- ○ Self Regional Healthcare
- ○ University of South Carolina

Pro00114653: Partial Heart Transplantation for Semilunar Heart Valve Dysfunction - Taufiek Rajab

View: Study Identification - IRB Review Request - v2

IRB Review Request for Multi-site Studies

The ***Reviewing IRB*** is the IRB with the primary responsibility for reviewing a study. is the IRB with the primary responsibility for reviewing a study. The single IRB (sIRB) mandate is a set of federal policies that require certain types of federally-funded studies that involve multiple institutions to use a single IRB to accomplish IRB review and approval for all participating sites. **If you are requesting one of these IRB reviews, indicate the type below. Otherwise, click Continue on this page.**

Effective Date: January 20, 2020

Applies to: Federally funded cooperative research projects receiving initial IRB approval on or after January 20, 2020. This pertains to studies that involve more than one institution conducting research even if institutions are performing different human subject research activities.

Reviewing IRB: Will be identified by the Federal department or agency supporting or conducting the research or proposed by the lead institution (subject to the acceptance of the Federal department or agency supporting the research).

IRB OF RECORD IS YOUR INTERNAL IRB

Single IRB (sIRB) Review (multi-site research reviewed by one IRB)

The institution that allows this in eIRB is MUSC & USC.

1.0 * **Is this a request for a single IRB review?**

Has your local IRB agreed to serve as the IRB of record for a multi-site study and at least 1 of those relying sites will have its own principal investigator.

Be sure to contact your local IRB and confirm they have agreed to serve as the IRB of record.

○ Yes ● **No**

IRB OF RECORD IS ANOTHER IRB

External IRB Review

2.0 External IRB Review means that your local/internal IRB has agreed to rely on another IRB for IRB review

* **Is this a request for your local IRB to rely on another IRB?**

○ Yes ● **No**

Be sure to contact your local IRB to assure that appropriate authorization agreements have been or will be executed.

Pro00114653: Partial Heart Transplantation for Semilunar Heart Valve Dysfunction - Taufiek Rajab

View: Study Identification - MUSC IRB Selection

MUSC Institutional Review Board Selection

1.0 * **Select the appropriate committee:**

○	IRB-I - Medical University of South Carolina	Cell and Molecular Pharmacology & Experimental Therapeutics; College of Health Professions; College of Nursing; College of Pharmacy; Dermatology; Harper Student Life Center; MUHA - Clinical/Surgical Services and Excellence, AHEC; Otolaryngology; Pathology and Laboratory Medicine; Pediatrics; Psychiatry and Behavioral Science; Radiology; Regenerative Medicine and Cell Biology; Urology; VA Pharmacy
●	**IRB-II - Medical University of South Carolina**	**Anesthesiology and Perioperative Medicine; Biochemistry and Molecular Biology; College of Dental Medicine; Emergency Medicine; Family Medicine; Library Sciences and Informatics; Medicine; Microbiology and Immunology; Neurology and Neurosurgery; Neurosciences; Obstetrics and Gynecology; Ophthalmology; Orthopaedics and Physical Medicine; Public Health Sciences; Radiation Oncology; Surgery**
○	IRB-III - Medical University of South Carolina	All Departments Industry Sponsored Trials Only

Pro00114653: Partial Heart Transplantation for Semilunar Heart Valve Dysfunction - Taufiek Rajab

View: Study Identification - Study Personnel Affiliation

Study Personnel Affiliation

1.0 * **Are all personnel on this research study affiliated with the institution of the internal IRB? If no, the next screen will contain a list of HSSC eIRB users for all institutions.**

● **Yes** ○ No

Pro00114653: Partial Heart Transplantation for Semilunar Heart Valve Dysfunction - Taufiek Rajab

View: Study Identification - Study Personnel (Institution Specific)

Study Personnel

1.0 * **Principal Investigator:**
Click the Select button and choose a PI
Taufiek Rajab

2.0 **Study Coordinator**
Click the Select button and choose the individual who will assist in coordinating the overall activities of the research study.

3.0 **Co-Investigator(s)**
Click the Add button and select the Co-Investigators for this study:
PI must obtain agreement of co-investigators prior to submitting their names to the study.

Name	Organization
John Costello	PEDIATRICS - MUSC
Minoo Kavarana	CARDIOTHORACIC SURGERY - MUSC

4.0 **Other Study Team Member(s)**
Click the Add button and select any other team members (other project assistants, students, etc.):

Name	Credentials	Organization	Role on Study	Edit Permission
Henderson, Heather Tripp M.D.	M.D.	PEDIATRICS - MUSC		yes
Savage, Andrew J MD	MD	CARDIOLOGY PEDS - MUSC		yes
Taylor, Carolyn MD	MD	CARDIOLOGY PEDS - MUSC		yes
Welch, Brett		Medical University of South Carolina		yes

5.0 **Guest List**
Click the Add button and select any user to have read-only access to study information:

Name	Credentials	Organization
Andrew Atz	M.D.	CARDIOLOGY PEDS - MUSC

Pro00114653: Partial Heart Transplantation for Semilunar Heart Valve Dysfunction - Taufiek Rajab

View: Study Identification - eIRB Communication Coordinators

eIRB Communication Coordinators

1.0 * **Select those study team members that will handle eIRB communication for this study.**

	Person
☑	**John Costello**
☐	Heather Henderson
☑	**Minoo Kavarana**
☑	**Taufiek Rajab**
☐	Andrew Savage
☐	Carolyn Taylor
☐	Brett Welch

Pro00114653: Partial Heart Transplantation for Semilunar Heart Valve Dysfunction - Taufiek Rajab

View: Study Identification - Study Sites

Study Sites

1. * **Indicate all affiliated sites that will be involved in the research study.**
Check all that apply:

- [x] MUSC
- [ ] VAMC
- [ ] SCTR Research Nexus (formerly CTRC)
- [ ] Hollings Cancer Center
- [ ] Investigational Drug Service (Investigator MUST contact Pharmacy Services 843-792-9643 to get information on requirements and budget)
- [ ] MUSC Health Chester Medical Center
- [ ] MUSC Health Florence Medical Center
- [ ] MUSC Health Lancaster Medical Center
- [ ] MUSC Health Marion Medical Center
- [ ] MUSC Health Kershaw Medical Center
- [ ] MUSC Health Columbia Medical Center
- [ ] MUSC Heart and Vascular Institute

List any other facilities where research activities will take place:

2. * **Will this study involve sites that meet ALL of the criteria listed below?**
1) Are not MUSC sites
2) Are hosting or conducting research procedures
3) Do not have their own Principal Investigator

○ Yes ● **No**

Pro00114653: Partial Heart Transplantation for Semilunar Heart Valve Dysfunction - Taufiek Rajab

View: Human Subjects Research - Human Subjects Research

Human Subjects Research

The following questions will assist you in determining whether this project meets the federal requirements for Human Subjects Research.

1.0 * **Is this project a systematic investigation, including research development, testing, and evaluation, designed to develop or to contribute to generalizable knowledge?**

● **Yes** ○ No

Reference: OHRP definition of Research

2.0 * **Does this project involve a living individual about whom an investigator conducting research:**
(i) Obtains information or biospecimens through intervention or interaction with the individual, and uses, studies, or analyzes the information or biospecimens; or
(ii) Obtains, uses, studies, analyzes, or generates identifiable private information or identifiable biospecimens?

● **Yes** ○ No

Reference: OHRP definition of Human Subject

3.0 **Does this project involve a Humanitarian Use Device (HUD)?** *A HUD is a device intended to benefit patients in the treatment or diagnosis of a disease or condition affecting not more than 8,000 individuals in the US per year. See additional guidance on HUD Designations.*

○ Yes ● **No**

Pro00114653: Partial Heart Transplantation for Semilunar Heart Valve Dysfunction - Taufiek Rajab

View: Training - CITI Training Records

CITI Training Records

Review this information when considering if human subjects research education/training is complete for all investigators and study staff. Personnel training displayed are the current and historical records required by the institution associated with the team member's eIRB user account.

NOTE: All study team members must be in compliance with training requirements prior to beginning any role in the study.

If training is missing or expired:
1. Instructions for completing research education requirements can be found at www.musc.edu/citi.
2. Verify this institution's affiliation has been added to the CITI user profile and complete the required training.
3. Verify the first name, last name and preferred email of the CITI user profile matches the eIRB user profile.

The content on this page is provided as a tool to display research training records in real time. These data are routinely updated and are, therefore, current at the present viewing of this content.

1.0 Principal Investigator CITI Completion Records

Name	Organization	Curriculum	Group	Stage	Date Earned	Date Expires
Taufiek Rajab	Medical University of South Carolina	Basic/Refresher Human Subjects Research Curriculum	Group 1. Biomedical Investigators and Key Personnel	Basic Course	7/18/2020	7/18/2023
		GCP Social and Behavioral Research Best Practices for Clinical Research	GCP Social and Behavioral Research Best Practices for Clinical Research	Basic Course	9/25/2020	9/25/2023
		Good Clinical Practice Course for Clinical Trials Involving Drugs (ICH focus)	GCP for Clinical Trials with Investigational Drugs and Biologics (ICH Focus)	Basic Course	9/25/2020	9/25/2023

(Columns Curriculum through Date Expires appear under the heading "Completed CITI Training".)

2.0 Study Coordinator CITI Completion Records

Name	Organization	Completed CITI Training

3.0 Co-Investigator(s) CITI Completion Records

Name	Organization	Completed CITI Training				
		Curriculum	Group	Stage	Date Earned	Date Expires
John Costello	Medical University of South Carolina	Basic/Refresher Human Subjects Research Curriculum	Good Clinical Practice and ICH	Refresher Course	8/2/2021	8/1/2024
		Basic/Refresher Human Subjects Research Curriculum	Good Clinical Practice and ICH	Basic Course	8/6/2018	8/5/2021
		Basic/Refresher Human Subjects Research Curriculum	Group 1. Biomedical Investigators and Key Personnel	Refresher Course	9/7/2021	9/6/2024
		Basic/Refresher Human Subjects Research Curriculum	Group 1. Biomedical Investigators and Key Personnel	Basic Course	10/20/2018	10/19/2021
Minoo Kavarana	Medical University of South Carolina	Basic/Refresher Human Subjects Research Curriculum	Good Clinical Practice and ICH	Refresher Course	10/4/2021	10/3/2024
		Basic/Refresher Human Subjects Research Curriculum	Good Clinical Practice and ICH	Basic Course	8/29/2018	8/28/2021
		Basic/Refresher Human Subjects Research Curriculum	Group 1. Biomedical Investigators and Key Personnel	Refresher Course	8/14/2020	8/14/2023
		Basic/Refresher Human Subjects Research Curriculum	Group 1. Biomedical Investigators and Key Personnel	Basic Course	3/25/2011	3/25/2014

4.0 Other Study Team Member(s) CITI Completion Records

Name	Organization	Completed CITI Training				
		Curriculum	Group	Stage	Date Earned	Date Expires
Heather Henderson	Medical University of South Carolina	Basic/Refresher Human Subjects Research Curriculum	Good Clinical Practice and ICH	Refresher Course	12/7/2020	12/7/2023
		Basic/Refresher Human Subjects Research Curriculum	Group 1. Biomedical Investigators and Key Personnel	Refresher Course	12/8/2020	12/8/2023
		Basic/Refresher Human Subjects Research Curriculum	Group 1. Biomedical Investigators and Key Personnel	Basic Course	11/20/2017	11/19/2020
		Good Clinical Practice Course for Clinical Trials Involving Drugs (ICH focus)	GCP for Clinical Trials with Investigational Drugs and Biologics (ICH Focus)	Basic Course	8/12/2022	8/11/2025
Andrew Savage	Medical University of South Carolina	Basic/Refresher Human Subjects Research Curriculum	Good Clinical Practice and ICH	Refresher Course	4/30/2020	4/30/2023
		Basic/Refresher Human Subjects Research Curriculum	Good Clinical Practice and ICH	Basic Course	3/24/2014	3/23/2017
		Basic/Refresher Human Subjects Research Curriculum	Group 1. Biomedical Investigators and Key Personnel	Refresher Course	4/30/2020	4/30/2023
		Basic/Refresher Human Subjects Research Curriculum	Group 1. Biomedical Investigators and Key Personnel	Basic Course	3/24/2014	3/23/2017

Name	Organization	Curriculum	Group	Stage	Date Earned	Date Expires
Carolyn Taylor	Medical University of South Carolina	Basic/Refresher Human Subjects Research Curriculum	Good Clinical Practice and ICH	Refresher Course	11/29/2021	11/28/2024
		Basic/Refresher Human Subjects Research Curriculum	Good Clinical Practice and ICH	Basic Course	2/22/2013	2/22/2016
		Basic/Refresher Human Subjects Research Curriculum	Group 1. Biomedical Investigators and Key Personnel	Refresher Course	12/21/2021	12/20/2024
		Basic/Refresher Human Subjects Research Curriculum	Group 1. Biomedical Investigators and Key Personnel	Basic Course	2/22/2013	2/22/2016
Brett Welch	Medical University of South Carolina	Basic/Refresher Human Subjects Research Curriculum	Group 1. Biomedical Investigators and Key Personnel	Basic Course	8/5/2021	8/4/2024
		Good Clinical Practice Course for Clinical Trials Involving Drugs (ICH focus)	GCP for Clinical Trials with Investigational Drugs and Biologics (ICH Focus)	Basic Course	8/5/2021	8/4/2024

5.0 Mentor CITI Completion Records

Name	Organization	Completed CITI Training

6.0 Collaborating Institutions PI(s) CITI Completion Records

Name	Organization	Completed CITI Training

There are no items to display

Pro00114653: Partial Heart Transplantation for Semilunar Heart Valve Dysfunction - Taufiek Rajab

View: Review Type - Study Review Type

Study Review Type

Minimal Risk means that the risks of harm anticipated in the proposed research are not greater -- considering probability and magnitude -- than those ordinarily encountered by the general population in daily life or during the performance of routine physical, laboratory, or psychological exams or tests.

1.0 * **Requested Review Type**

Select the type of IRB review you are requesting.

	Name	Description
○	Exempt	Research activities that present no risk or less than minimal risk as defined by the federal regulations 46.104
○	Expedited	Research activities that (i) present no more than minimal risk to human subjects, and (ii) involve only procedures listed in one or more of the categories authorized by 45 CFR 46.110 and 21 CFR 56.10
●	**Full IRB Review**	**The probability and magnitude of harm or discomfort anticipated in the research are greater in and of themselves than those ordinarily encountered in daily life or during the performance of routine physical or psychological examinations or tests**

Pro00114653: Partial Heart Transplantation for Semilunar Heart Valve Dysfunction - Taufiek Rajab

View: Protocol - Study Protocol

Study Protocol

1.0 * **Select the category that applies to your research study protocol:**

Name
○ Corporate/industry generated protocol for industry sponsored study
○ Protocol generated by Cooperative Group (national cancer groups, etc.)
○ Protocol from Federal grant application
● **Investigator generated protocol/research plan**
○ Dissertation or thesis proposal

2.0 **Protocol document, grant application, or research protocol**

* Click the Add button to upload these document(s). (**Note: Do NOT upload consent or any other documents here.**)

Name	Version	Orig. Author	Orig. Created	Last Modified
Protocol	0.09	Taufiek Rajab	12/11/2021 2:49 PM	4/21/2022 12:31 PM

3.0 **<u>Protocol Summary Information (as applicable):</u>**

3.1 **Study Protocol Version:** 2 **Dated:** 4/21/2022

3.2 **Sponsor Assigned Amendment Number:** **Dated:**

Pro00114653: Partial Heart Transplantation for Semilunar Heart Valve Dysfunction - Taufiek Rajab

View: Study Populations - Study Subjects

Study Subjects

1.0 * **Estimated Local Enrollment Goal**
Enter the anticipated number of subjects to be enrolled at local site:
5

2.0 **Estimated Study-Wide Enrollment Goal**
Enter the anticipated number of subjects to be enrolled at all sites:
5

3.0 * **Briefly describe the setting in which the research will be conducted.**
The interventional portion of the study will take place in the cardiac operating rooms. Observational data will be collected in the pediatric cardiac intensive care unit and the pediatric cardiac stepdown unit at MUSC Shawn Jenkins Children's Hospital, and in the MUSC Health outpatient pediatric cardiology clinics.

4.0 * **Participant Remuneration (Payment/Academic Credit)**
Will subject(s) receive remuneration?
○ Yes ● **No**

5.0 * **Will prospective participants be vulnerable to coercion or undue influence?**
○ Yes ● **No**

If yes, briefly describe additional safeguards included in the protocol to protect the rights and welfare of participants likely to be vulnerable.

6.0 * **Identify targeted subject population(s) involved in this research study** (**Note:** The purpose of this question is to determine equitable selection of subjects and to identify vulnerable populations.)

a. Check the following included population(s) to illustrate the equity of subject population selection. Select all that apply:

- [x] **Males**
- [x] **Females**
- [x] **Minorities**

b. Check the following included population(s) to identify populations that require additional protections. Select all that apply:

- [ ] Pregnant Women
- [x] **Human Fetuses or Neonates**
- [x] **Children**

- [ ] Prisoners
- [ ] Persons with impaired decision making capacity
- [ ] Employees of an investigators' institution
- [ ] Students enrolled at the principal investigator's institution
- [x] **Non-English speaking persons**
- [x] **Economically, socially, or educationally disadvantaged persons**

7.0 * **Study Population**
Briefly describe the study population? (e.g. healthy volunteers, adults with Type II Diabetes, children with Asthma):
Children less than 2 years of age who require semilunar heart valve replacement

8.0 * **Describe the selection criteria (inclusion/exclusion criteria):**
Inclusion Criteria
• Children less than 2 years of age who are referred for a cardiac operation that involves a primary semilunar valve replacement or children less than 2 years of age who are referred for a cardiac operation involves an initial replacement of a previously placed prior homograft, bioprosthetic, or mechanical valve in the aortic or pulmonary position.
• Deemed acceptable for partial heart transplantation based on the standard evaluation process used for orthotopic heart transplantation (see Appendix 1)
• Insurance approval.
• Written informed consent of both parents/guardians; if there is only one parent/guardian, consent from that individual will be adequate.

Exclusion Criteria
• Absolute contraindications for orthotopic heart transplantation.
Severe bilateral long segment pulmonary arterial hypoplasia
Bilateral pulmonary vein stenosis
<34 weeks corrected gestational age
Persistent acidosis with a pH < 7.1
• Diagnosis of immune deficiency.
• Inability for the parent to understand English.
• Failure to pass the following psychosocial evaluation:
o The candidate should reside within 4 hours traveling time from Medical University of South Carolina for a minimum of four to six months post-transplantation to assure careful follow-up
o The candidate's family should be capable of long-term supportive care of the child and be able to support the medical needs of the child in follow-up
o Parental (custodial) alcohol and/or substance abuse
o Documented parental (custodial) child abuse or neglect
o Parent (custodian) with cognitive/psychiatric impairment severe enough to limit comprehension of medical regimen
• Infectious Disease Exclusion Criteria
o Evidence of sepsis

o Hepatitis B surface antigenemia
o HIV positivity

9.0 * **Describe recruitment procedures, including how subjects will be contacted, by whom, and how eligibility will be determined.**

A study investigator who normally participates in our biweekly heart center case conference meeting and has access to patient records and surgical referrals for valve replacement will identify potential candidates for the trial.
For outpatients, when feasible, an introductory letter, study summary, invitation to discuss the study by phone, and "opt out" card will be mailed to parents/guardians several weeks prior to the date of surgery. Telemedicine may be utilized to initially discuss the risks and benefits associated with the study with parents/guardians of potential subjects who are outpatients, but an in-person meeting will be required for a formal consent discussion.

Mothers who are carrying fetuses that are expected to require heart surgery requiring a valve replacement within the first 2 years of life may be approached for enrollment in the study.

Once permission has been obtained from the primary cardiologist and/or cardiac surgeon, a study investigator will approach the parents / guardians of eligible patients to discuss the study, including potential risks and benefits, and seek written, informed consent. These discussions will occur in the preoperative clinic on the third floor of Shawn Jenkins Children's Hospital. For eligible patients who are hospitalized in the pediatric CICU or Cardiac Stepdown Unit in the days preceding surgery, discussions will take at place at the bedside or any consult room.

The parents/guardians of all subjects who were approached by study investigators will also meet with an attending pediatric cardiologist or cardiac surgeon who is not involved with the study to discuss conventional surgical valve replacement options and anticipated outcomes.

For subjects to be enrolled in the trial, a time window will be established by an attending cardiologist not involved with the study as to how long the patient can be listed for partial heart transplantation before a conventional valve replacement option is scheduled. This time window will be determined patient based on the patient's clinical status and severity of valve dysfunction and may be revised over time based on evolving clinical status of the patient.

10.0 * **Will cold contact be used as a method of recruitment?**

○ Yes ○ No

Pro00114653: Partial Heart Transplantation for Semilunar Heart Valve Dysfunction - Taufiek Rajab View: Study Populations - Socially/Economically Disadvantaged Persons

Socially/Economically Disadvantaged Persons

1.0 Describe the safeguards that are included in this study to protect the rights and welfare of the Socially/Economically Disadvantaged Persons who may be included in this study.

Subjects will be assured that the study is voluntary and that their standard of care will not be impacted if they choose not to participate. They will also be told that they may withdraw at any time if they no longer want to continue with the trial.

Pro00114653: Partial Heart Transplantation for Semilunar Heart Valve Dysfunction - Taufiek Rajab

View: Study Population - Research Directed Toward Pregnant Women

Research Directed Toward Pregnant Women

1.0

The research activities must present minimal risk to the fetus, or be designed to meet the health needs of the mother, and the fetus will be placed at risk only to the minimum extent necessary to meet such needs.
Explain how the research satisfies this requirement.
Only mothers who are carrying fetuses that are expected to require heart surgery requiring a valve replacement within the first 2 years of life may be considered. There will be no harm to the fetus as there will be no procedures performed on the fetus or mother. Only after the baby is born and is in need of valve replacement, will a procedure be performed and only if the proper consent is received.

2.0 **Explain what measures will be taken to ensure that any risk to the fetus beyond that necessary to meet the health needs of the fetus or the mother is minimal and is the least possible risk for achieving the objectives of the study.** (Note: When applicable, appropriate studies on animals and non-pregnant individuals must have been completed.)
The study will be discussed in a way and an environment that minimizes stress for the mother.

3.0

Will individuals engaged in the research also be involved in decisions as to the timing, method, or procedures used to terminate a pregnancy or determine the viability of the fetus?
○ Yes ● **No**

If YES, explain:

4.0

Will informed consent be obtained from both the mother and father?
● **Yes** ○ No

If NO, provide an explanation.

Pro00114653: Partial Heart Transplantation for Semilunar Heart Valve Dysfunction - Taufiek Rajab

View: Study Population - Research Directed Toward Fetuses

Research Directed Toward Fetuses

1.0

* **Does the research study involve fetuses in utero?**

○ Yes ● **No**

If YES, the research activity must be designed to meet the health needs of the fetus and the fetus will be placed at risk only to the minimum extent necessary to meet such needs, or the research must present minimal risk to the fetus and the purpose of the activity is to obtain important knowledge that cannot be obtained by other means.

Explain how the research satisfies this requirement:

2.0

* **Does the research involve fetuses ex utero?**

○ Yes ● **No**

If yes, the research activity must present no added risk to the fetus and the purpose of the activity is the development of important knowledge that cannot be obtained by other means, or the purpose of the activity is to enhance the possibility of survival of the fetus to the point of viability.

No nonviable fetus may be involved as a subject unless; vital functions of the fetus will not be artificially maintained, the research activities which of themselves would terminate the heartbeat or respiration of the fetus will not be employed, and the purpose of the research activity is the development of important knowledge which cannot be obtained by other means.

Explain how the research satisfies these requirements:

3.0

* **Will informed consent be obtained from both the mother and father?**

● **Yes** ○ No

If NO, explain:

Pro00114653: Partial Heart Transplantation for Semilunar Heart Valve Dysfunction - Taufiek Rajab

View: Study Population - Non-English Speaking Subjects

Non-English Speaking Subjects

1.0 **If the research will routinely include subjects who speak a language other than English, the informed consent documents should be translated into that language. Please indicate the language(s) and method of translation.**

The consents will be translated into Spanish by a translation service.

2.0 **Describe the credentials of the translator(s).**

Translators' Credentials:

Click the Add button to upload the credentials of the translator(s).

Name	Version	Orig. Author	Orig. Created	Last Modified

There are no items to display

3.0 **Describe the process to be used to explain the research study and assure that the non-English speaking subjects will understand the study and their participation in research.**

Clinical certified translators will be used to explain the research study and assure that the non-English speaking subjects will understand the study and their participation in research.

Pro00114653: Partial Heart Transplantation for Semilunar Heart Valve Dysfunction - Taufiek Rajab

View: Funding and Sponsorship - Study Funding Information

Study Funding Information

1.0 * **Primary Funding Source (Active or Pending)**

Select primary (active or pending) funding sources for this study:

- [ ] Federal Government
- [ ] Private Industry
- [ ] Private Not-for-Profit Organization
- [ ] State or Local Government
- [x] **Internal Funding**
- [ ] Non-US Funding
- [ ] Other
- [ ] No Funding

Pro00114653: Partial Heart Transplantation for Semilunar Heart Valve Dysfunction - Taufiek Rajab

View: Funding and Sponsorship - Study Sponsorship

Study Sponsorship

1.0 Sponsor(s)
Click the Add button and select the sponsor(s)

Name

There are no items to display

2.0 Other Sponsor(s)
If sponsor(s) is/are not in the above list above, enter name(s) here:

3.0 External Identifier (if applicable):
(e.g. agency/sponsor assigned numbers)

4.0 Internal Identifier (if applicable):
(e.g. proposal or award Number)

Pro00114653: Partial Heart Transplantation for Semilunar Heart Valve Dysfunction - Taufiek Rajab

View: Funding and Sponsorship - IIT

Internally Sponsored or Unsponsored Research

1.0 PAYING UDAK

Please enter the paying UDAK for the IRB fee. ?

Entity:	Account:	Unit:	Project:	Reporting:	**If Entity is MUCR, Enter Project Year** (a sequential number representing the current grant year [i.e., 01, 02, etc.]):
* MUCU	50228	* 2270200	* 39726	* 9525	

UDAK:MUCU502282270200397269525

2.0 IIT Number (Optional)

An IIT is not necessary; however, you may enter an IIT number along with the UDAK.

Pro00114653: Partial Heart Transplantation for Semilunar Heart Valve Dysfunction - Taufiek Rajab

View: Funding and Sponsorship - Study Costs

Study Costs

Study Associated costs funded by:

1.0 Drug(s):

- [ ] N/A
- [ ] Sponsor
- [ ] Participant
- [x] **Other**

If OTHER, specify:
The patients receive the same drugs as they would for a conventional heart transplant. Therefore this is expected to be covered by the patient's insurance. If the patient's insurance does not cover the costs then the patient won't be eligible for a partial heart transplant and will instead require a conventional heart transplant.

2.0 Device(s):

- [x] **N/A**
- [ ] Sponsor
- [ ] Participant
- [ ] Other

If OTHER, specify:

3.0 Supplies:

- [x] **N/A**
- [ ] Sponsor
- [ ] Participant
- [ ] Other

If OTHER, specify:

4.0 Laboratory Fees:

- [ ] N/A

- [ ] Sponsor
- [ ] Participant
- [x] **Other**

If OTHER, specify:
The patients receive the same laboratory tests as they would for a conventional heart transplant. Therefore this is expected to be covered by the patient's insurance. If the patient's insurance does not cover the costs then the patient won't be eligible for a partial heart transplant and will instead require a conventional heart transplant.

5.0 Hospital Fees:

- [ ] N/A
- [ ] Sponsor
- [ ] Participant
- [x] **Other**

If OTHER, specify:
The patients receive the same hospital fees as they would for a conventional heart transplant. Therefore this is expected to be covered by the patient's insurance. If the patient's insurance does not cover the costs then the patient won't be eligible for a partial heart transplant and will instead require a conventional heart transplant.

6.0 Physician Fees:

- [ ] N/A
- [ ] Sponsor
- [ ] Participant
- [x] **Other**

If Other, specify:
The patients receive the same physician fees as they would for a conventional heart transplant. Therefore this is expected to be covered by the patient's insurance. If the patient's insurance does not cover the costs then the patient won't be eligible for a partial heart transplant and will instead require a conventional heart transplant.

7.0 Other:

- [x] **N/A**

- [ ] Sponsor
- [ ] Participant
- [ ] Other

If OTHER, specify:

Pro00114653: Partial Heart Transplantation for Semilunar Heart Valve Dysfunction - Taufiek Rajab

View: Checklist - Application Checklist

Application Checklist

1.0 Will the following be involved in the research study?
Select all that apply

Subject Consent Documentation/Authorization

- [ ] Deception (Requires Waiver or Alteration of Informed Consent and debriefing procedures)
- [x] **Informed consent document(s)**
- [ ] Waiver of the Requirement to Obtain Written and Signed Consent
- [ ] Waiver or alteration of informed consent procedure or elements

Data Capture/Review/Monitoring/Storage

- [ ] Data from the statewide Health Sciences South Carolina (HSSC) Clinical Data Warehouse ?
- [x] **Data Safety Monitoring Plan**
- [x] **Medical Record, Chart Review**
- [ ] The storage of biological specimens (e.g. biological material, tissue, blood, etc.) for potential future, yet undesignated, research
- [ ] The storage of Data (e.g. subject level data, images, scans, recordings, etc.) for potential future, yet undesignated, research
- [ ] Use of survey, questionnaire, focus group, interviews, group discussion

Recruitment

- [ ] Advertisements or recruiting materials

Laboratory/Specimens

- [x] Specimens (blood, urine, tissue and other human products)
- [ ] Human pluripotent or embryonic stem cells (including their use and/or derivation) ?

- [x] ### Drugs/Chemicals/Substances

Drugs, chemicals, metabolites, nutritional substances, biological agents or other substances whether regulated or not by the Food & Drug Administration (FDA) that will be administered to subjects

- [ ] Use of Placebos

Devices

- [ ] Medical devices, instruments, machines, computer programs or other device, including Humanitarian Use Devices (HUDs), whether FDA approved or not

Radiation

- [ ] Diagnostic or therapeutic ionizing radiation, or radioactive isotopes that are not part of clinical standard of care

Biosafety/Genetics

- [ ] Human Genetic Research
- [ ] Human In Vitro Fertilization
- [ ] Recombinant or synthetic nucleic acid molecules, gene transfer, infectious agents, select agents or microorganism, or biological toxin (including Botulinum toxins)
- [x] **Transplantation**
- [ ] Vaccines

Research Populations

- [ ] Cancer-related research ?
- [ ] Healthy, normal volunteers as research subjects
- [ ] Individuals with HIV/AIDS as research subjects
- [ ] This research study will be conducted by you or your research personnel at other institutions/sites outside of the US
- [x] **Clinical trial**

Research Themes

- [ ] Alcohol and Drug Abuse Research

Pro00114653: Partial Heart Transplantation for Semilunar Heart Valve Dysfunction - Taufiek Rajab

View: Checklist - Data and Safety Monitoring Plan (DSMP)

Data and Safety Monitoring Plan (DSMP)

1.0 * **Provide a general description of the data and safety monitoring plan:**

The study investigators will be primarily responsible for monitoring all study patients for the occurrence of adverse events and unanticipated problems as outlined above.
A data safety and monitoring board (DSMB) will be used to monitor the progress of this clinical trial and review safety and effectiveness data while the trial is ongoing.

2.0 * **Is there a data and safety monitoring board/committee (DSMB/DSMC) to review for safety and adherence to the study protocol?**

● **Yes** ○ No

3.0 **If yes, describe the composition of the board/committee and their qualifications:**

A physician will chair the DSMB. Members will be comprised of senior faculty from other institutions, and will include a pediatric transplant cardiologist, a pediatric cardiac intensivist, and a pediatric cardiac surgeon. None of these individuals will be directly involved with the undertaking of this study or affiliated with MUSC.

4.0 **Describe the frequency of DSMB/DSMC reviews and reports, planned interim analysis, etc.**

The DSMB will be responsible for reviewing the research protocol (and any amendments thereof) and the informed consent documents prior to the onset of patient recruitment. The DSMB will meet virtually 1 month after each of the first 3 patients are enrolled and annually thereafter to review study data. The study investigators will prepare the interim reports, including a summary of outcomes and adverse events, for the DSMB to review.

5.0 **Describe plans for assuring compliance with requirements regarding the reporting of Unanticipated Problems Involving Risks to Participants or Others and/or Adverse Events to the IRB and appropriate regulatory agencies.**

The DSMB will review major protocol modifications proposed by the study team; participant recruitment, accrual and retention; confidentiality issues; consider factors external to the study when relevant, such as scientific or therapeutic developments that may have an impact on the safety of the subjects or the ethics of the trial; and review the investigator-assigned relationships between the intervention and adverse events. Following each meeting, the DSMB will prepare a written report on the safety and scientific progress of the trial and make recommendations to the PI concerning continuation, termination or modification of the trial based on the observed beneficial or adverse effects of the research. The DSMB will also meet at the conclusion of the study, and as needed to review serious adverse events.
A subject will be removed from the study if the parent/guardian withdraws consent, or if one of the patient’s primary attending physicians elects to remove the patient from the study. Additionally, all clinicians who are uninvolved with the trial but participate in the care of the subjects will be encouraged to report adverse events to the study investigators. The study investigators will meet every six months to review adverse events and to

assess the risk/benefit ratio for continuing the trial.
Any action that results in a temporary or permanent suspension of the trial will be reported as soon as possible by the investigators to the MUSC IRB and DSMB. If the DSMB or IRB requests a temporary or permanent suspension of the trial, they will notify the principle investigator who will promptly notify all coinvestigators. Annually, the principal investigator and study coordinator will meet for data verification and protocol compliance checks. Specifically, these individuals will monitor whether all enrolled subjects met entry criteria, whether any deviations from the study protocol occurred, and whether any patients were prematurely removed from the protocol by managing physicians or withdrawal of parental consent. The principal investigator or study coordinator will review all CRFs and data within the study database for completeness and accuracy on an annual basis.

Pro00114653: Partial Heart Transplantation for Semilunar Heart Valve Dysfunction - Taufiek Rajab

View: Other Study Specifics - Clinical Trials

Clinical Trials

1.0 * **What is the phase of the clinical trial?**

- [ ] Phase I clinical trial
- [ ] Phase II clinical trial
- [ ] Phase III clinical trial
- [ ] Phase IV clinical trial
- [ ] Symptom Management
- [ ] Prevention Trial
- [ ] Observational
- [ ] Interventional
- [x] **Other**
- [ ] This study does not involve a clinical trial
- [ ] Open-Label Extension Study

2.0 **If OTHER, describe:**
pilot study

Pro00114653: Partial Heart Transplantation for Semilunar Heart Valve Dysfunction - Taufiek Rajab

View: Other Study Specifics - Study Procedures

Study Procedures

(Blood draw, Imaging, Lab Tests, Physical Exam, Medical History)

1.0 * **Briefly describe the procedures to be performed solely as part of this research study.**

A chart review will be performed. A donor heart will be procured and the donor heart valve will be removed from the donor heart. The actual implantation of this donor valve (i.e. the partial heart transplant) is the same as for a homograft valve replacement (standard of care). Post-operatively, the patient will be treated with immunosuppression.

2.0 * **Briefly describe the procedures being performed already for diagnostic or treatment purposes (standard of care).**

Procedures being performed already as standard of care are clinic visits, diagnostic labs, echocardiography, cardiac catheterization, and cross-sectional imaging.

Pro00114653: Partial Heart Transplantation for Semilunar Heart Valve Dysfunction - Taufiek Rajab

View: Other Study Specifics - Study Risks and Precautions

Study Risks and Precautions

1.0 * **Risks, Discomforts and Potential Harms**

Briefly describe the risks associated with all aspects of the study. Include consideration of physical, psychological, social, financial, and other factors, as applicable.

In conventional heart transplantation, the most serious risks are graft failure, cardiac allograft vasculopathy, infection and acute rejection. Together these account for the majority of deaths after conventional heart transplantation. The relative importance of these causes of death vary over time post-transplant. There are also scheduling issues related to donor heart availability, which require patients and parents to be alerted to arrive at the hospital with limited notice.

The risks for a partial heart transplantation are lower than orthotopic heart transplantation. There is no risk for transplant coronary artery disease or myocardial dysfunction. We expect that graft failure and cardiac allograft vasculopathy will not be important risks after partial heart transplantation. The reason for this is that conventional heart transplants perform active work to sustain hemodynamics, whereas the partial heart transplant merely serves a structural function. There is no need for repeat cardiac catheterizations with myocardial biopsy for invasive assessment of hemodynamics and surveillance of rejection and transplant coronary artery disease. There is no risk of tricuspid regurgitation due to tricuspid valve apparatus damage from recurrent myocardial biopsies.

Risk of death: we expect the operative risk of partial heart transplantation to be similar to the risks from homograft valve replacement. We expect the long-term risks to be lower than the long-term risks from conventional heart transplantation and far lower than the long-term risks from re-operations for homograft implant exchanges.

Risks of partial heart transplant:

Rejection of transplanted valve

Immunosuppression risks in partial heart transplantation are similar to the immunosuppression risks in conventional heart transplantation
• Daily burden of taking medication
• Infection, especially bacterial infections.
• Side effects for each medication
o Tacrolimus
o CellCept
o Corticosteroids
• Outpatient lab draws
• Compliance- should be very good for infants/young children

Additional outpatient follow-up:
• Frequency of outpatient heart transplant clinic follow-up visits

Risk of loss of confidentiality.

Other potential risks:

Data collected as part of the study protocol will be available only to study investigators. Confidentiality will be respected and maintained at all times. The investigators will obtain permission from the primary cardiologist or cardiac surgeon to speak with parents/guardians about the study to

protect their privacy.

Pediatric cardiac surgical procedures result in significant parental anxiety and distress. The study investigators are familiar interacting with parents in this situation and will be cognizant of parental needs. No aspects of this study are expected to have a deleterious effect on parents' psychological well-being.

The risks for a partial heart transplantation are controllable and overall lower than orthotopic heart transplantation.

2.0 * **Describe the safety precautions that will be taken to minimize risks/harms. This should include your data protection management plan:**

Intraoperative risks will be controlled by performing the operation in large animals for training.
Postoperative risks will be controlled by follow-up of the patients using the tried and tested protocols for conventional heart transplant recipients.

Data collected as part of the study protocol will be available only to study investigators. Confidentiality will be respected and maintained at all times. The investigators will obtain permission from the primary cardiologist or cardiac surgeon to speak with parents/guardians about the study to protect their privacy.

Pediatric cardiac surgical procedures result in significant parental anxiety and distress. The study investigators are familiar interacting with parents in this situation and will be cognizant of parental needs. No aspects of this study are expected to have a deleterious effect on parents' psychological well-being.

3.0 * **Provide a plan for close monitoring of subjects by qualified clinicians, including review of adverse events, incidental findings, and follow up of safety assessments, as applicable.**

Subjects will be monitored by clinicians qualified in pediatric heart transplantation cardiology. All clinicians who are uninvolved with the trial but participate in the care of the subjects will be encouraged to report adverse events to the study investigators. The study investigators will meet every six months to review adverse events and incidental findings to assess the risk/benefit ratio for continuing the trial.

Pro00114653: Partial Heart Transplantation for Semilunar Heart Valve Dysfunction - Taufiek Rajab View: Other Study Specifics - Potential Benefits

Potential Benefits

Benefit - A research benefit is considered to be something of health-related, psychosocial, or other value to an individual research subject, or something that will contribute to the acquisition of generalizable knowledge. Money or other compensation for participation is generally not considered to be a research benefit.

1.0 * **Benefit Category**

Select a benefit Category:

- ○ This research study is not expected to directly benefit individual subjects, but is likely to yield generalizable knowledge which contributes to the field.
- ● **This research study involves the prospect of direct benefit to the individual subject.**

2.0 * **What are the potential benefits of the research study to the subject and/or to society?**

Explain:

• The most important advantage of partial heart transplantation is growth of the transplanted valve. This minimizes the morbidity and mortality for patients from successive implant exchanges.
• Avoiding reoperations will also improve neurodevelopmental outcomes as repeat cardiac operations are associated with worse neurodevelopmental outcomes (Fuller et al. Predictors of impaired neurodevelopmental outcomes at one year of age after infant cardiac surgery. European Journal of Cardio-Thoracic Surgery 2009).
• Avoiding reoperations will decrease psychological and financial stress for the families.
• The transplanted valve would also be durable without the need for anticoagulation and heal minor injuries from mechanical trauma or infection.
• Better valve function with more favorable loading conditions on the ventricular myocardium compared to mechanical valve replacements.
• Potential longer longevity.

Other benefits:
Parents may benefit from knowing that the scientific knowledge gained from this study may be of benefit to society and future patients.

Risk/benefit assessment:
The investigators believe that partial heart transplantation is superior to the alternative treatment options available to all patients (e.g. homograft valve replacement) and equipoise exists with the remaining alternative treatment options available to a subset of patients (e.g. Ross procedure) to conduct the proposed study. The potential risks of participation in this trial are outweighed by the potential benefits.

Pro00114653: Partial Heart Transplantation for Semilunar Heart Valve Dysfunction - Taufiek Rajab

View: Conflict of Interest - Conflict of Interest

Conflict of Interest

***Definition* - Conflict of Interest**: conflict of interest means that because of activities or relationships with other persons or organizations, an individual is unable, or potentially unable, to remain impartial, that the individual's objectivity is, or might be otherwise impaired, or that the individual has, or might acquire, an unfair competitive advantage. Information that is relevant to a conflict of interest determination includes stock holdings and investments of the individual, the individual's spouse or dependent children; current positions held or under negotiation; any other sources of income; involvement in the design, conduct, or reporting of the research and any other relevant information that may have a bearing on the individual's proposed participation.

***Definition*- Financial Interest Related to the Research:** means financial interest in the sponsor, product or service being tested, or competitor of the sponsor or product or service being tested.

1.0 * **Do any of the participating study investigators or other research personnel (or their immediate family) have a financial and/or intellectual property interest in the sponsor or products used with this research study?**

○ Yes ● **No**

Pro00114653: Partial Heart Transplantation for Semilunar Heart Valve Dysfunction - Taufiek Rajab

View: Consent Process - Consent Process

Consent Process

1. * **Will the consent be obtained from the subject?**
   ○ Yes ● **No**

2. **Will the consent be obtained from the subject's legally authorized representative?**
   ● **Yes** ○ No

3. **Describe any waiting period between informing the prospective participant and obtaining consent:**
   There is no required waiting period between informing the subject's legally authorized representative about the study and then obtaining consent.

4. * **Who will obtain consent?**
   Please list research personnel authorized and qualified to obtain informed consent
   T. Konrad Rajab, M.D.
   Minoo N. Kavarana, M.D.
   John Costello, M.D.

5. * **Will electronic consent be used for this study?**
   ○ Yes ● **No**

6. * **Describe the consent process (where, when and how a written copy of the consent form will be provided).**
   Once permission has been obtained from the primary cardiologist and/or cardiac surgeon, a study investigator will approach the parents or guardians of eligible patients to discuss the study. The parents will be given a full description of all the treatment options for their child, including the experimental valve transplant procedure, and those who are interested in the study will then be given a copy of the consent form to participate in this study. Parents will be given time to read the consent in its entirety and have all of their questions answered before agreeing to have their child participate. Written informed consent will then be obtained. Both parents will be asked to consent if both parents are available to do so.

7. **Consent Forms**

   To allow for documentation of IRB approval and electronic watermarking, please use the following link to access your institution's Informed Consent Form Template:

   Template

   https://research.musc.edu/resources/ori/irb/forms

   If no template is available, please leave at least a **one inch margin at the bottom of each page** of the final "clean" version of the consent document(s).

   NOTE: When revising a consent document associated with an amendment or continuing review, Click "Upload Revision" to upload the

revised version of the consent document. Use ADD only when uploading a new consent document.

* Click the Add button to upload a copy of the consent form(s), including translated versions for this research study.

Name	Version	Orig. Author	Orig. Created	Last Modified
Consent Form	0.08	Taufiek Rajab	12/11/2021 2:51 PM	4/21/2022 12:30 PM

Pro00114653: Partial Heart Transplantation for Semilunar Heart Valve Dysfunction - Taufiek Rajab

View: Consent Process - Children as Participant

Children as Participant

Legally, a child is an individual who has not yet reached his or her eighteenth (18) birthday, unless the individual is or has been legally married, or has been emancipated by a court of law. The marriage certificate or a copy of the court order should be requested as documentation to confirm emancipated child (minor) status. Consent for a child to participate in research must be provided by a parent or legal guardian who is judged by the person administering obtaining consent to be able to understand and make health care decisions. Parent is defined as "a child's biological or adoptive parent" and guardian as "an individual who is authorized under applicable State law to consent on behalf of a child to general medical care".

1.0 * **Research involving children must be assigned to a permissible risk category. Choose the category that applies to this research study:**

- ○ The research does not involve greater than minimal risk to the individual subjects
- ● **The study involves greater than minimal risk to the subjects but presents the prospect of direct benefit to the individual subjects**
- ○ The study involves greater than minimal risk to the subjects and presents no prospect of direct benefit to the individual subjects, but is likely to yield generalizable knowledge about the subject's disorder or condition
- ○ The research is not otherwise approvable but presents an opportunity to understand, prevent, or alleviate a serious problem affecting the health or welfare of children.

  Note: approval by the Secretary of Health & Human Services (HHS) is required if the research is supporteed by HHS.

NOTE: Minimal risk means that the risks of harm anticipated in the proposed research study are not greater -- considering probability and magnitude -- than those ordinarily encountered in daily life or during the performance of routine physical, laboratory, or psychological exams or tests.

2.0 * **Provide justification for assigned risk category:**

The subjects have a diagnosis that requires a heart surgery for heart valve replacement at a very young age, <2 years old. The diagnosis will cause the initial risk and the need for heart surgery will also causes risk. From there the patient's guardians are required to make a decision for the patient on which surgical option they wish to choose (e.g. homograft valve replacement, Ross procedure, orthotopic heart transplantation). We are providing an alternative option, for which the risks are expected to be less than orthotopic heart transplantation.

Pro00114653: Partial Heart Transplantation for Semilunar Heart Valve Dysfunction - Taufiek Rajab

View: Privacy - Privacy and Confidentiality

Privacy and Confidentiality

1.0 * **Describe the procedures and safeguards that will be implemented to protect the privacy and confidentiality of the participants' data. Include details, as applicable to the study, such as: privacy of interview site, procedures for coding/de-identifying data and the extent to which identifiable private information is or has been de-identified and the risk that such de-identified information can be re-identified, the potential risk of harm to individuals should the information be lost, stolen, compromised, or otherwise used in a way contrary to the contours of the research under the exemption, the extent to which the information will be shared or transferred to a third party or otherwise disclosed or released, the security controls that are in place to protect the confidentiality and integrity of the information; the likely retention period and persons with access to private identifiable data, etc.**

The following procedures and safeguards will be implemented to protect the privacy and confidentiality of the participants' data:

* The interview site will be private.
* All completed CRFs will remain confidential and will be stored in locked cabinets within the locked offices of the study investigators at MUSC, accessible only to study investigators.
* Study data will reside in password protected computers within locked offices of study investigator at MUSC, accessible only to study investigators.
* All data stored on MUSC servers are password protected and backed up nightly. * The network is protected on multiple levels that include firewalls, intrusion detection software as well as continuous monitoring by the Information Services Security Department.
* The will not be shared or transferred to a third party or otherwise disclosed or released.
* Research subject identifiers will be destroyed three years after enrollment is completed.

2.0 * **Where will study records and data collected at this site be stored**
Select all that apply:

- [x] **In a locked office**
- [x] **In a locked cabinet**
- [x] **Password protected network storage**
- [ ] Password protected end-user/portable device (desktop computer, laptop, palm pilot, blackberry, etc.)
- [ ] Other

If OTHER, describe:

If information will be stored on an end-user/portable device, describe the security on the end-user/portable device that will be used to prevent unauthorized access to the data in the event the device is lost or stolen:

3.0 * **Will the study use a National Institutes of Health (NIH) Certificate of Confidentiality?**

○ Yes ● **No**

If YES, what is the NIH Certificate of Confidentiality status?

○ The NIH Certificate of Confidentiality has been approved

○ Will apply for a NIH Certificate of Confidentiality for this study

4.0 **Research Documentation in the Legal Medical Record**

Any research project where research documentation will occur in MUSC's electronic medical record Epic should include appropriate, relevant language in the research informed consent document available as part of the Informed Consent template

Please select one of the following options:

● **Study participation WILL be documented in Epic (research procedures will be ordered, scheduled or billed through Epic, and/or the study will have an immediate impact on clinical care)**

○ Study participation will NOT be documented in Epic

Link to MUSC policy for Research Documentation in the Legal Medical Record

Pro00114653: Partial Heart Transplantation for Semilunar Heart Valve Dysfunction - Taufiek Rajab

View: Privacy - Protected Health Information (PHI) for Research

Protected Health Information (PHI) for Research

Protected Health Information (PHI) is defined as individually identifiable health information transmitted or maintained in any form (electronic means, paper, or oral communication) that relates to the past, present, or future physical or mental health or conditions of an individual.

Covered Entity - A health plan, a health care clearinghouse, or a health care provider who transmits health information in electronic form.

1.0 * **To determine if this research study is using/disclosing PHI, select any of the following 18 elements that your study will require access to, as defined by the Health Insurance Portability and Accountability Act (HIPAA), as identifiers. If none of these 18 identifiers will be used/disclosed, then select the final option.**

If the study requires access to any of the 18 identifiers but will not be linked to PHI, please select those that are applicable to the left. On the next screen, you will be able to select "Study where health information is not linked to identifiers".

- [x] **Names**
- [x] **All geographic subdivision smaller than a state including street address, city, county, precinct, zip code, and/or equivalent geocodes.**
- [x] **All elements of date (except year) for dates directly related to an individual (DOB, admission date, discharge date, date of death)**
- [x] **Telephone numbers**
- [ ] Fax numbers
- [ ] Electronic mail addresses
- [ ] Social security numbers
- [x] **Medical record number**
- [ ] Health plan Beneficiary number
- [ ] Account numbers
- [ ] Certificate/license numbers
- [ ] Vehicle Identifiers and serial numbers, including license plate numbers
- [ ] Device identifiers and serial numbers
- [ ] Web Universal Resource Locators (URLs)
- [ ] Internet Protocol (IP) address numbers
- [ ] Biometric identifiers, including finger and voice prints

- [ ] Full face photographic images and any comparable images
- [ ] Any other unique identifying number, characteristic or code
- [ ] *None of the above 18 identifiers will be used/disclosed for this research study*

Pro00114653: Partial Heart Transplantation for Semilunar Heart Valve Dysfunction - Taufiek Rajab

View: Privacy - Access to Protected Health Information (PHI) for Research

Access to Protected Health Information (PHI) for Research

1.0 Indicate the sources of health information to be used
Select all that apply:

- [x] **Medical Records/Physician Notes/Hospital Discharge Records**
- [ ] Psychotherapy Notes
- [x] **Medical Test Results**
- [x] **Payment/Billing/Insurance records**
- [ ] Biological samples obtained from subjects for non research purposes
- [ ] Databases/Registries
- [ ] Tissue Repositories
- [ ] Other

If OTHER, indicate any other source(s) of health information to be collected/used:

2.0 How will PHI be accessed for the research study? (Check all those that apply)

- [x] **HIPAA Research Authorization**
- [ ] HIPAA Waiver of Authorization for Research
- [ ] Accessing Information for Preparatory Work for Research
- [ ] Accessing Information Through Limited Data Sets
- [ ] Accessing Deceased Persons’ Information
- [ ] Access Information through De-Identification
- [ ] Study where health information is not linked to identifiers

Pro00114653: Partial Heart Transplantation for Semilunar Heart Valve Dysfunction - Taufiek Rajab

View: Privacy - HIPAA Research Authorization

HIPAA Research Authorization

An authorization is an individual's signed permission to allow a covered entity to use or disclose the individual's protected health information (PHI) that is described in the authorization for the purpose(s) and to the recipient(s) stated in the authorization. A valid authorization for research must contain core elements and required statements as specified by 45 CFR 164.508(c)(1) & (2).

1.0 Click on the following link to access the HIPAA Research Authorization Form.

https://research.musc.edu/resources/ori/irb/forms

Upload HIPAA Research Authorization Form:

Name	Version	Orig. Author	Orig. Created	Last Modified

There are no items to display

2.0 * Summarize the procedures to be used when obtaining authorization:

HIPAA will be obtained via the combined ICF/HIPAA document.

Pro00114653: Partial Heart Transplantation for Semilunar Heart Valve Dysfunction - Taufiek Rajab View: Privacy - Use of De-Identification to Access Protected Health Information (PHI)

Use of De-Identification to Access Protected Health Information (PHI)

1.0 The Privacy Rule permits covered entities, depending on IRB approval, to use and disclose data that have been de-identified without obtaining an authorization. The Principal Investigator must receive the data from the covered entity in a de-identified format. A covered entity may de-identify PHI in one of two ways. The first method requires the removal of every one of 18 identifiers enumerated at section 164.514(b)(2) of the Privacy Rule. Data that are stripped of these 18 identifiers are regarded as de-identified. The second way to de-identify PHI is to have a qualified statistician determine, using generally accepted statistical and scientific principles and methods, that the risk is very small that the information could be used, alone or in combination with other reasonably available information, by the anticipated recipient to identify the subject of the information.

The Privacy Rule permits a covered entity to assign to, and retain with, the health information a code or other means of record identification if that code is not derived from or related to the information about the individual and could not be translated to identify the individual. The covered entity may not use or disclose the code or other means of record identification for any other purpose and may not disclose its method of re-identifying the information.

* **Will you be receiving de-identified information from a covered entity for the purpose of this project?**

○ Yes ● **No**

Pro00114653: Partial Heart Transplantation for Semilunar Heart Valve Dysfunction - Taufiek Rajab

View: Drugs - Drugs

Drugs

* **1.0 Indicate which of the following will be involved in this research study**
Select all that apply:

Marketed drugs will be used in this research study

2.0 Drug Management Plan

If you have not already done so, go to the **Medication Management Algorithm** and follow the instructions at the end of the form. Then, return to eIRB and answer the following question based on the form's results.

* **Did you receive one of the following recommendations from the Medication Management Plan Algorithm?**
"Based on your responses above, your medication management plan is appropriate for your study."
"Since you will not be obtaining medications for research purposes you do not need to continue with this form."

● **Yes** ○ No

* **2.a. Medication Management plan**
Click on the Add button to upload applicable document(s)

Name	Version	Orig. Author	Orig. Created	Last Modified
Medication Management Plan	0.01	Taufiek Rajab	1/7/2022 8:18 PM	1/7/2022 8:18 PM

Pro00114653: Partial Heart Transplantation for Semilunar Heart Valve Dysfunction - Taufiek Rajab

View: Drugs - Marketed - Part 1

Drugs - Marketed (Part 1)

1.0 * **List all marketed drugs to be used in the study:**
Tacrolimus, mycophenolate, methylprenisolone

8.0 **Investigator's Brochure or Package Insert:**
Click the Add button to upload the Investigator's brochure or Package insert document(s):

Name	Version	Orig. Author	Orig. Created	Last Modified
Cellcept	0.01	Taufiek Rajab	1/10/2022 9:31 AM	1/10/2022 9:31 AM
Methylprednisolone	0.01	Taufiek Rajab	1/10/2022 9:31 AM	1/10/2022 9:31 AM
Tacrolimus	0.01	Taufiek Rajab	1/10/2022 9:31 AM	1/10/2022 9:31 AM

Pro00114653: Partial Heart Transplantation for Semilunar Heart Valve Dysfunction - Taufiek Rajab

View: Drugs - Marketed - Part 2

Drugs - Marketed (Part 2)

2.0

* **Is each drug approved by the FDA for this indication?**

● **Yes** ○ No

If NO, list drug(s):

3.0

* **Is the route of administration approved by the FDA?**

● **Yes** ○ No

If NO, list drug(s):

4.0

* **Is the use for this study patient population approved by the FDA?**

● **Yes** ○ No

If NO, list drug(s):

5.0

* **Is the dosage level approved by the FDA?**

● **Yes** ○ No

If NO, list drug(s):

6.0 **Where will drugs be stored?**

7.0

Where will drugs be dispensed?

Pro00114653: Partial Heart Transplantation for Semilunar Heart Valve Dysfunction - Taufiek Rajab

View: Drugs – Other Substances

Drugs – Other Substances

1.0 **List chemicals, metabolites, nutritional substances, biological agents or other substances.**

2.0

Is an IND anticipated for the use of this substance in this research study?

○ Yes ○ No

Explain:

Pro00114653: Partial Heart Transplantation for Semilunar Heart Valve Dysfunction - Taufiek Rajab View: General Comments

General Comments

1.0 Add any additional comments to assist in the review of this research study.

2.0 Add any miscellaneous documents that do not fit in other sections of the study application.

Click Add to upload document(s)

Name	Version	Orig. Author	Orig. Created	Last Modified

There are no items to display

Pro00114653: Partial Heart Transplantation for Semilunar Heart Valve Dysfunction - Taufiek Rajab

View: SCresearch.org Directory

SCresearch.org Directory

SCresearch.org is a web based research studies directory designed to promote research opportunities available within the HSSC Consortium. Use of this directory is appropriate for any research study currently recruiting human subjects. Specific information from this eIRB application will be used to populate the directory

This study will automatically be included in the directory unless you un-check the box below.

☐ **INCLUDE** this study on the SCresearch.org website

Pro00114653: Partial Heart Transplantation for Semilunar Heart Valve Dysfunction - Taufiek Rajab

View: Research Master ID (RMID) - v2

Research Master ID (RMID)

A Research Master ID (RMID) is a unique number that links the study among MUSC research systems by associating common data such as PI, long/short protocol titles, funding source and department. An RMID is required for study applications and is created on the MUSC Research Master ID website, which also includes details about this process.

Enter the Research Master ID (RMID) associated with this study. *If the study's RMID is not known, CLICK HERE to go to the RMID website to search or create one. Then, return here to add the RMID to this study application.*

* **Research Master ID:**
6732

Pro00114653: Partial Heart Transplantation for Semilunar Heart Valve Dysfunction - Taufiek Rajab

View: SF - Final Page

Final Page

This is the end of the application.

Select the **'Finish'** button to exit this page. If you are the Principal Investigator, you may select on the **'Submit'** activity in the Application workspace to send to the IRB Office.
