# Supplementary material for: Partial heart transplantation for pediatric heart valve dysfunction: A clinical trial protocol
Source: PLoS One. 2023 Feb 7;18(2):e0280163. doi: 10.1371/journal.pone.0280163 (PMC9904480; doi:10.1371/journal.pone.0280163)
Supplement: Attachment — Submitted filename: Response to Reviewers.docx [file pone.0280163.s006.docx]

**Comments to the Author**

1. Does the manuscript provide a valid rationale for the proposed study, with clearly identified and justified research questions?

The research question outlined is expected to address a valid academic problem or topic and contribute to the base of knowledge in the field.

Reviewer #1: Yes

Reviewer #2: Yes

2. Is the protocol technically sound and planned in a manner that will lead to a meaningful outcome and allow testing the stated hypotheses?

The manuscript should describe the methods in sufficient detail to prevent undisclosed flexibility in the experimental procedure or analysis pipeline, including sufficient outcome-neutral conditions (e.g. necessary controls, absence of floor or ceiling effects) to test the proposed hypotheses and a statistical power analysis where applicable. As there may be aspects of the methodology and analysis which can only be refined once the work is undertaken, authors should outline potential assumptions and explicitly describe what aspects of the proposed analyses, if any, are exploratory.

Reviewer #1: Yes

Reviewer #2: Partly

3. Is the methodology feasible and described in sufficient detail to allow the work to be replicable?

Descriptions of methods and materials in the protocol should be reported in sufficient detail for another researcher to reproduce all experiments and analyses. The protocol should describe the appropriate controls, sample size calculations, and replication needed to ensure that the data are robust and reproducible.

Reviewer #1: Yes

Reviewer #2: Yes

4. Have the authors described where all data underlying the findings will be made available when the study is complete?

The [PLOS Data policy](https://journals.plos.org/plosone/s/materials-and-software-sharing) requires authors to make all data underlying the findings described in their manuscript fully available without restriction, with rare exception, at the time of publication. The data should be provided as part of the manuscript or its supporting information, or deposited to a public repository. For example, in addition to summary statistics, the data points behind means, medians and variance measures should be available. If there are restrictions on publicly sharing data—e.g. participant privacy or use of data from a third party—those must be specified.

Reviewer #1: Yes

Reviewer #2: No

5. Is the manuscript presented in an intelligible fashion and written in standard English?

PLOS ONE does not copyedit accepted manuscripts, so the language in submitted articles must be clear, correct, and unambiguous. Any typographical or grammatical errors should be corrected at revision, so please note any specific errors here.

Reviewer #1: Yes

Reviewer #2: Yes

6. Review Comments to the Author

Please use the space provided to explain your answers to the questions above and, if applicable, provide comments about issues authors must address before this protocol can be accepted for publication. You may also include additional comments for the author, including concerns about research or publication ethics.

You may also provide optional suggestions and comments to authors that they might find helpful in planning their study.

(Please upload your review as an attachment if it exceeds 20,000 characters)

Reviewer #1: Ochoa and colleagues describe a protocol for a prospective nonrandomized, single-center, single arm pilot trial where a “partial heart transplant” will be performed in infants and young children with semilunar valve disease. The hypothesis is that the transplant semilunar valves will grow with the patient and avoid known complications associated with current valve options for these children. This a reasonable protocol, however, I have following specific comments for the authors

“Inability for the parent(s) or legal guardian(s) to understand English or Spanish”, this seems like an inappropriate exclusion criterion, especially when all study procedures are standard clinical care, and one would expect that inability to understand these languages does not preclude clinical care.

RESPONSE: This exclusion criterion was added because we do not have the ability to translate the clinical trial consent form accurately into other languages.

There is another ethical issue/potential risk with this procedure that is not discussed. A potential organ for life-saving heart transplant is diverted for a treatment that has other surgical options. This should be discussed and potentially be part of the informed consent. Furthermore, donor selection is not discussed in detail, could these donors have suboptimal cardiac function and not suitable for conventional heart transplant?

RESPONSE: We agree that donor selection is important and needs to be discussed. We also agree that donors with suboptimal cardiac function and not suitable for conventional transplant would be ideal. We added the following information to the discussion: “Partial heart transplantation can be performed using donor hearts with poor ventricular function and slow progression to donation after cardiac death. This should ameliorate donor heart utilization and avoid both primary orthotopic heart transplantation in children with unrepairable heart valve dysfunction and progression of these children to end-stage heart failure.” We also cited a recent review that we wrote about this very important topic (Sherard et al. Partial heart transplantation can ameliorate donor organ utilization. J Card Surg . 2022 Oct 19. doi: 10.1111/jocs.17050. Online ahead of print.).

It may be after this submission, but there has been a clinical case described as “first partial heart transplant” from Duke University in the news lately. Citation of that would be important for complete reference of prior work.
RESPONSE: this case is not yet published in the scientific literature.

Lower levels of immunosuppression are proposed as a possibility after first year, a description of such protocol is important for completeness.

RESPONSE: This is beyond the scope of the current clinical trial. Therefore, the reference to lower levels of immunosuppression was deleted.

Reviewer #2: This study addresses an interesting topic. It is well detailed and may lead to interesting results. Some comments follow.

1. The sample size is rather small. It is necessary to specify the power of the study. Similarly, the fact that it is a nonrandomized single-center study may strongly limit its practical usefulness. Indeed, my main concern is about how general the obtained results would be. It is difficult to understand why such a small sample size is aimed to be collected.

RESPONSE: The reason for the small sample size is the cost of the operation. Partial heart transplantation involves procurement of the donor heart using jet airplane transport in order to minimize the ischemia time. Therefore a larger number of patients for this pilot trial would be prohibitive.

2. Missing data management needs a better discussion. Missing data could be completely-at-random, at-random, not-at-random. Sensitivity analysis should be planned to avoid misleading inference whenever complete-case analysis is performed.
RESPONSE: We agree. The following paragraph was inserted in the data management methods section: “Missing data will be addressed by omission or analysis as appropriate. Sensitivity analysis will be planned to avoid misleading inference when complete-case analysis is performed.”

3. The data analysis is based on descriptive statistics only. This strongly limits the project. If research questions cannot be tested, if inference cannot be performed, the study simply describes a specific sample, that may not be representative of a larger population. Which general info do you expect to get from a simple descriptive analysis? Cause-effect relationships cannot be investigated, nor any characterization of the outcomes could be discussed. The data analysis is not sufficient to provide informative results. I strongly suggest to plan a more detailed and extensive statistical analysis, based on a larger sample. Statistical inference must be guranteed.

RESPONSE: We agree that a more detailed extensive statistical analysis based on a larger sample would be helpful. We are planning this as the next step once funding for can be secured, either for insurance companies that already fund all expenses for conventional heart transplants or via a grant. The following sentence was added to the conclusion: “In the future, extensive statistical analysis based on a larger sample will be necessary to further evaluate partial heart transplantation.”
